# YOLO Object Detection for Real-Time Fabric Defect Inspection in the Textile Industry: A Review of YOLOv1 to YOLOv11

**DOI:** 10.3390/s25072270

**Published:** 2025-04-03

**Authors:** Makara Mao, Min Hong

**Affiliations:** 1 Department of Software Convergence, Soonchunhyang University, Asan-si 31538, Republic of Korea; makaramao07@gmail.com; 2 Department of Computer Software Engineering, Soonchunhyang University, Asan-si 31538, Republic of Korea

**Keywords:** YOLO variants, real-time defect detection, fabric detection, deep learning in textiles, convolutional neural networks, textile industry, quality control

## Abstract

Automated fabric defect detection is crucial for improving quality control, reducing manual labor, and optimizing efficiency in the textile industry. Traditional inspection methods rely heavily on human oversight, which makes them prone to subjectivity, inefficiency, and inconsistency in high-speed manufacturing environments. This review systematically examines the evolution of the You Only Look Once (YOLO) object detection framework from YOLO-v1 to YOLO-v11, emphasizing architectural advancements such as attention-based feature refinement and Transformer integration and their impact on fabric defect detection. Unlike prior studies focusing on specific YOLO variants, this work comprehensively compares the entire YOLO family, highlighting key innovations and their practical implications. We also discuss the challenges, including dataset limitations, domain generalization, and computational constraints, proposing future solutions such as synthetic data generation, federated learning, and edge AI deployment. By bridging the gap between academic advancements and industrial applications, this review is a practical guide for selecting and optimizing YOLO models for fabric inspection, paving the way for intelligent quality control systems.

## 1. Introduction

Fabric quality control is a critical component of the textile and garment manufacturing industry, where defects such as tears, stains, holes, and misaligned patterns can compromise the aesthetic and functional value of the final product. Traditionally, fabric inspection has relied on human operators, whose ability to detect subtle defects depends on experience, intuition, and judgment [1]. However, human inspection is inherently limited by factors such as lethargy, inconsistency, and high labor costs [2]. This makes it unsuitable for modern high-speed production lines where efficiency and precision are paramount [3,4]. Recent advances in computer vision, particularly You Only Look Once (YOLO) models, have transformed quality control by enabling real-time, high-accuracy detection of fabric defects. Traditional manual fabric inspection is labor intensive and prone to inconsistencies, making it ineffective for modern high-speed production lines. The adoption of deep learning-based automated defect detection systems has accelerated the resolution of these challenges [5].

Among various automated approaches, object detection models powered by deep learning have shown remarkable potential in revolutionizing quality control processes in textile manufacturing [6]. Specifically, the YOLO family of models has emerged as a leading framework for real-time object detection. YOLO’s single-stage detection framework is highly advantageous for the textile industry, where high-speed production lines necessitate efficient and scalable defect detection systems [7]. YOLO-based systems provide consistent defect detection performance across shifts and varying production volumes, thereby eliminating human error and ensuring scalability in high-throughput environments [8,9]. The YOLO family of models has emerged as a leading real-time object detection framework, offering a highly efficient and scalable solution for fabric defect detection in high-speed manufacturing environments [10]. Despite these advantages, applying YOLO models in fabric defect detection presents several challenges, including handling diverse defect types, varying fabric textures, and lighting conditions while maintaining high precision and speed [11,12].

YOLO has evolved from its early speed-focused iterations from YOLO-v1 to YOLO-v3 to more advanced architectures from YOLO-v4 to YOLO-v11 that integrate attention mechanisms, multi-scale detection, and feature fusion to improve defect localization and classification [13]. In contrast, recent versions incorporate advanced features such as Transformer-based attention mechanisms, multi-scale detection, and adaptive learning, significantly enhancing the ability to detect subtle and complex defects in fabrics [14,15]. These advancements align with the growing adoption of Industry 4.0 practices, where automation and real-time analytics are integral to maintaining competitiveness in global markets. The findings of this review have significant industrial implications, including enabling faster defect detection, reducing production downtime, and lowering costs [16], thereby driving efficiency in real-world manufacturing processes.

Although several surveys have addressed the application of deep learning in fabric defect detection [17], most prior works provide a limited scope, focusing either on traditional techniques or specific YOLO versions and application scenarios [18]. For example, Rasheed et al. [19] reviewed traditional histogram- and texture-based methods. Similarly, Czimmermann et al. [20] provided a detailed taxonomy of defects. However, they noted the absence of a universal detection method for diverse materials. In contrast, Li et al. [21] and Ahmad et al. [22] highlighted the shift toward learning-based methods, but they lacked coverage of recent architectural innovations, such as Transformer integration. More recently, Neethu et al. [23] compared traditional and automated defect detection methods, but their discussion on the challenges and limitations of deep learning models in real-word industrial settings was limited.

In contrast, this review comprehensively analyzes YOLO models, ranging from YOLO-v1 to YOLO-v11, with a focus on their architectural evolution and real-world applications in fabric defect detection. Unlike earlier reviews that addressed traditional methods or a limited subset of YOLO versions, this study systematically evaluates the performance, strengths, and limitations of the entire YOLO family, specifically in the context of textile manufacturing. By incorporating recent advancements such as attention-based mechanisms, multi-scale detection, and Transformer integration, we provide an in-depth evaluation of how each YOLO version contributes to addressing challenges in fabric defect detection, including defect variability, texture adaptation, robustness to lighting conditions, and computational efficiency. This broader perspective bridges the gap between academic development and industrial application, serving as a practical guide for researchers and practitioners in selecting and optimizing YOLO models for fabric inspection tasks.

### 1.1. Survey Objective

The primary objective of this research is to evaluate the evolution of YOLO variants and their applications in fabric defect detection using YOLO models, addressing the growing need for accurate and efficient quality control in textile manufacturing. This review seeks to answer the following key research questions:

1. Effectiveness of YOLO Models: How do YOLO-based models compare to traditional inspection methods in detecting and localizing fabric defects, and which specific defect types are best identified using these models?
2. Integration with Advanced Architectures: How do innovations like Transformer integration and attention mechanisms enhance the accuracy, speed, and adaptability of YOLO models in real-world manufacturing environments?
3. Challenges and Industrial Adoption: What are the critical challenges in deploying YOLO-based systems for fabric defect detection, including dataset diversity, real-time performance, and scalability for high-speed production lines?

To address these research questions, this review presents a comprehensive analysis of YOLO object detection frameworks, ranging from YOLO-v1 to YOLO-v11, with a specific focus on their application in real-time fabric defect inspection within the textile industry. This paper systematically examines the architectural improvements and practical implications of each YOLO variant, highlighting their contributions to defect localization, accuracy, and industrial scalability. The key contributions of this review include the following:

- Providing an in-depth comparative analysis of YOLO models specifically designed for fabric defect detection tasks.
- Discussing the impact of recent innovations, including CIoU, CloU, attention mechanisms, and adaptive learning.
- Identifying current challenges and limitations in industrial deployment.
- Providing practical guidelines for model selection and future research directions in real-time fabric inspection systems.

The findings of this review highlight a research gap for future research and development efforts in fabric quality inspection and control, as well as the automated detection of various fabric types and advancements in YOLO variants. By leveraging the power of YOLO and its variants, the full potential for fabric defect detection can be realized, paving the way for new research directions and concentrated efforts in this domain.

### 1.2. Organization of Paper

**Figure 1:**
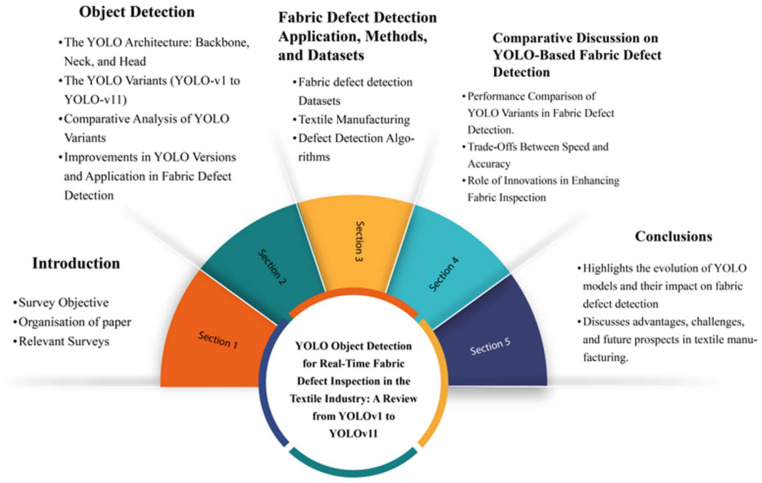
The overall structure of this study.

This paper is organized into the following sections: Section 2 presents the object detection and the original YOLO algorithm, followed by Section 2.2.1, Section 2.2.2, Section 2.2.3, Section 2.2.4, Section 2.2.5, Section 2.2.6, Section 2.2.7, Section 2.2.8, Section 2.2.9, Section 2.2.10 and Section 2.2.11, which cover the YOLO series from YOLO-v1 to YOLO-v11. Section 3 focuses on fabric defect detection using YOLO, applications in textile manufacturing, and fields where fabric defect detection and related applications can be utilized. Section 4 discusses fabric detection, highlighting the benefits of using YOLO for defect detection in industrial settings. Finally, Section 5 provides the conclusions, summarizing the paper’s key points and offering a conclusive evaluation of YOLO’s potential and limitations in the context of fabric defect detection. Figure 1 represents the review structure of this paper.

### 1.3. Relevant Surveys

Several prior surveys have explored fabric defect detection approaches, as outlined in Table 1. However, many of these studies primarily focus on limited subsets of YOLO models or general object detection techniques without thoroughly addressing the evolution and comparative performance of newer YOLO variants. Additionally, most existing works do not reflect the rapid advancements in deep learning architectures, such as the integration of attention mechanisms, Transformer modules, and advanced loss functions in YOLO-v9 to YOLO-v11. Moreover, few reviews systematically examine the practical trade-offs between detection accuracy, speed, and computational efficiency in real-time textile inspection scenarios. To address these gaps, this paper presents a comprehensive and up-to-date review of YOLO-based fabric defect detection, encompassing recent innovations and the challenges associated with industrial deployment.

Rasheed et al. [19] investigated traditional techniques, such as histogram-based and texture-based methods, but highlighted their sensitivity to conditions like lighting and imaging systems, which may affect reliability. Similarly, Czimmermann et al. [20] provided a detailed taxonomy of defects but noted the absence of a universal detection method for diverse materials. Li et al. [21] and Ahmad et al. [22] emphasized advancements in traditional and learning-based algorithm studies. They acknowledged challenges such as adapting methods to varying conditions and the high cost of data annotation.

Neethu et al. [23] and Jha et al. [24] demonstrated the efficiency of automated and deep CNN-based approaches. However, their reliance on large, labeled datasets and issues with model interpretability remain significant drawbacks. Kulkarni et al. [25] focused on proactive quality control through real-time monitoring, though the image quality constrained their system’s performance. Carrilho et al. [26] reviewed a broad spectrum of methodologies but noted the absence of standardized datasets for training and validation. Hussain [27] reviewed all major YOLO versions from v1 to v8, providing a detailed analysis of their architectural evolution, training strategies, and performance metrics. However, the application selection may not fully represent all possible uses of YOLO.

All these surveys emphasize the importance of fabric defect detection, and further research is necessary to address challenges such as the development of frameworks for object detection tools, the detection of complex fabric defects in real-world manufacturing environments, the integration of emerging deep learning models, and the improvement of scalability. In contrast, this study utilizes the YOLO framework for real-time defect detection, enabling the immediate identification of fabric defects and thereby enhancing the quality control processes in manufacturing.

## 2. Object Detection

Object detection is a fundamental task in computer vision that identifies and localizes objects within images. Recent advancements have significantly improved the accuracy and efficiency of deep learning, making it a key area of research [27]. Object detection involves identifying and localizing objects (e.g., humans, vehicles) within images. Over the past two decades, there has been a rapid advancement in object detection technology, which has profoundly influenced the entire field of computer vision [28].

In addition to fabric defect detection, object detection techniques have found widespread applications across various domains. In autonomous driving, object detection models are employed to identify vehicles, pedestrians, traffic signs, and obstacles, contributing to the development of intelligent transportation systems. In the healthcare field, object detection aids in identifying anomalies in medical imaging, such as tumors in CT scans and X-rays, enhancing diagnostic accuracy. Moreover, video surveillance systems utilize object detection to monitor suspicious activities in real time, supporting security and law enforcement efforts. These models aid in detecting pests, estimating crop yields, and monitoring plant health in agriculture. Object detection also plays a crucial role in retail, particularly for shelf inventory monitoring and customer behavior analysis, as well as in robotics and intelligent manufacturing, where it is used for visual inspection, object grasping, and defect classification. These cross-domain applications highlight the versatility and impact of object detection frameworks, such as YOLO, in addressing real-world problems across various sectors. Accuracy is essential in object detection, encompassing both classification and localization precision, as well as processing speed, two of the most critical metrics for evaluating object detection systems.

As a foundational task in computer vision, object detection supports many other applications, including instance segmentation [29], image captioning [30], and object tracking [31]. The rapid advancements in deep learning techniques over the past few years have significantly accelerated progress in object detection, resulting in remarkable breakthroughs and establishing it as a key area of research. Recently, object detection has been applied in various real-world scenarios, including autonomous driving [32], robotic vision [33], and video surveillance [34]. Among the most influential techniques in deep learning for object detection is the YOLO framework, which has demonstrated exceptional performance in detecting objects in both images and videos. Introduced by Fukushima [35] in 1980, convolutional neural networks (CNNs) underpin modern computer vision tasks, including activity recognition, image classification, text recognition, face recognition, and object detection and localization [36,37,38,39,40,41], as well as image characterization [42]. CNNs consist of interconnected neurons, each with learnable weights and biases, organized into layers—an input layer, multiple hidden layers (e.g., convolutional, pooling, and fully connected layers), and an output layer. The convolutional layer applies convolution operations to extract features such as edges or textures, while the pooling layer reduces spatial dimensions to prevent overfitting and enhance computational efficiency [43]. Fully connected layers integrate these features to make predictions. Inspired by the human visual cortex, CNNs mimic the hierarchical processing of visual stimuli, where lower layers detect basic features and higher layers recognize complex patterns.

Several studies have focused on developing efficient and robust approaches to fabric defect detection. Mak et al. [44] proposed a computationally efficient and robust fabric defect detection scheme that utilizes Gabor wavelets for feature extraction and morphological filters for defect isolation. The system’s performance was validated through both offline and real-time testing, demonstrating its effectiveness and potential for practical application in the textile industry. Their study employed a combination of a Gabor Wavelet Network (GWN) and morphological filters on a dataset comprising 78 fabric images (39 defect-free and 39 with defects) sourced from a standard defect manual. The results demonstrated an overall detection rate of 97.4%, a false alarm rate of 2.6%, and only two missed detections. The PRC-Light YOLO model proposed by Liu et al. [45] is a modified YOLO-v7 architecture designed for efficient and accurate detection of fabric defects.

The modifications focus on reducing computational costs while improving feature extraction and the robustness of the training process. The authors demonstrate the effectiveness of their approach through ablation studies and comparisons with other state-of-the-art object detection models. They replace YOLO-v7’s Single-Point Perfection and Complexity-Sensitive Perfection (SPPCSP) feature pyramid in the Neck module with the RFB. RFB employs multi-branch convolutions with varying dilation rates to capture multiscale information and expand the receptive field, thereby enhancing the detection of small defects. Jing et al. [46] present a practical and effective improvement to the YOLO-v3 model for fabric defect detection. Integrating optimized anchor boxes and enhanced feature fusion significantly improves detection accuracy, making this work a valuable contribution to the field of automated textile quality control. While the original YOLO-v3 model relies on pre-defined anchor boxes, which is not optimal for the varying sizes and shapes of fabric defects, they address the limitation by applying the K-means clustering algorithm to a labeled fabric defect dataset to determine the optimal number and sizes of anchor boxes—12 in this case—and improve the accuracy of bounding box regression.

**Figure 2:**
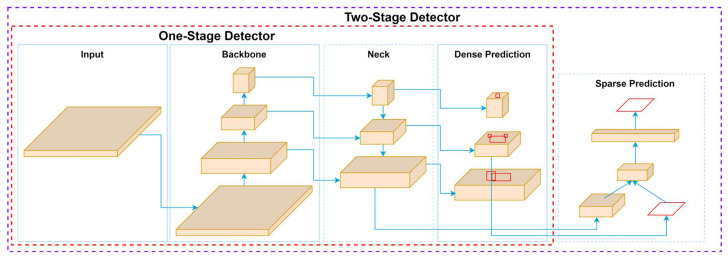
Object detector.

In object detection, CNNs are the foundation for single-stage (YOLO, SSD) and two-stage (Faster R-CNN) detectors, enabling object classification and image localization, as illustrated in Figure 2. Single-stage detectors integrate object classification and localization into a unified network, allowing for high-speed detection suitable for real-time applications. These detectors bypass intermediate stages, such as region proposal generation, and directly predict bounding boxes and class probabilities. While this design ensures efficiency, it may compromise accuracy, especially for small or occluded objects. On the other hand, two-stage detectors, such as Faster R-CNN [47], generate regional proposals and refine these proposals in a second stage for classification and localization [48]. This additional step improves precision but often comes at the cost of increased computational complexity and slower inference times. The choice between single-stage and two-stage detectors depends on the application’s speed and accuracy requirements, highlighting the versatility of CNNs in adapting to diverse object detection challenges.

Two-stage detectors, exemplified by Faster R-CNN [49], adopt a more segmented process to prioritize accuracy. The model generates regional proposals in the first stage, which are then classified and refined in the second stage. This approach allows two-stage detectors to achieve higher localization and classification precision [50]. However, the additional computational steps increase processing time, making them less suitable for real-time applications. Instead, two-stage detectors are ideal for scenarios where accuracy is critical, such as medical imaging [51], where detecting minute anomalies is vital, or industrial inspections, where detailed object recognition is required. The primary distinction between these approaches lies in their tradeoff between speed and precision. Single-stage detectors excel in scenarios where speed is paramount, while two-stage detectors are preferred for tasks that demand high accuracy and detailed object recognition.

### 2.1. The YOLO Architecture: Backbone, Neck, and Head

The YOLO architecture comprises three primary components—backbone, neck, and head—each of which undergoes significant modifications across its variants to enhance performance [52]. The backbone is responsible for extracting features from input data and typically consists of a convolutional neural network (CNN) that is pre-trained on large datasets, such as ImageNet [53]. Popular backbones in YOLO variants include ResNet-50, ResNet-101, and CSPDarkNet-53, each offering varying depth and feature extraction capabilities to balance speed and accuracy [54]. The neck further processes and refines the feature maps generated by the backbone. It often employs advanced techniques, such as Feature Pyramid Networks (FPNs) and Spatial Attention Modules (SAMs), to enhance feature representation and ensure robust object detection across different scales [55]. The head takes the fused features from the neck and predicts bounding boxes and class probabilities. YOLO’s head typically utilizes multi-scale anchor boxes, enabling effective detection of objects across a wide range of sizes [56]. This multi-scale approach ensures that YOLO performs well in detecting both small and large objects in diverse environments.

### 2.2. The YOLO Variants

**Figure 3:**
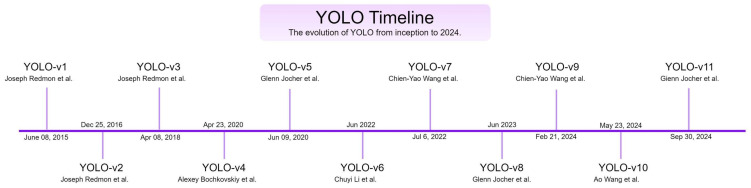
Evolution of the YOLO versions [57,58,59,60,61,62,63,64].

YOLO is a real-time object detection framework designed to localize and classify objects in images efficiently. Object detection, a key task in computer vision, leverages neural networks to achieve these objectives. This section focuses on the evolution of YOLO variants from YOLO-v1 to YOLO-v11, as illustrated in Figure 3. The foundation of all YOLO models lies in CNNs, which are widely used in object detection tasks. Researchers and engineers have adopted YOLO models for object detection and segmentation applications due to their open source nature and versatility.

#### 2.2.1. YOLO-v1: The Original

**Figure 4:**
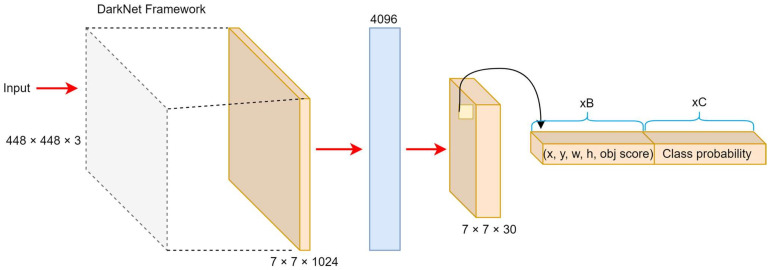
The Architecture of YOLO-v1.

YOLO-v1, introduced by Redmon et al. [57], was the first real-time object detection model to treat detection as a single regression problem. It divides the input image into a grid, with each cell predicting bounding boxes and class probabilities in one forward pass. Based on the Darknet framework, this architecture enables real-time processing with impressive speed, as illustrated in Figure 4. YOLO-v1 processes 448 × 448 images through a convolutional network, generating a 7 × 7 × 1024 feature map, followed by fully connected layers that produce a 7 × 7 × 30 output. Each cell predicts bounding boxes with position, confidence score, and class probabilities.

Its loss function includes localization, confidence, and classification components, using penalties to prioritize accurate object detection and suppress false positives. However, due to its coarse grid structure, it struggles with small or adjacent objects and exhibits higher localization errors compared to two-stage detectors, such as Faster R-CNN [44]. Despite these limitations, YOLO-v1’s fast inference of 45 FPS with 63.4% mAP on the PASCAL VOC dataset made it highly suitable for time-sensitive applications [65]. A faster variant, “Fast YOLO”, achieved 155 FPS at the cost of reduced accuracy (52.7% mAP).

#### 2.2.2. YOLO-v2

YOLO-v2, introduced by Redmon and Farhadi in 2016 [58], is an improvement over the original YOLO model, which was developed for real-time object detection with enhanced accuracy and speed. It addresses the critical limitations of YOLO-v1 by refining its architecture and introducing new techniques. Specifically, YOLO-v2 enhances object detection tasks and improves classification by incorporating interactive improvements, such as batch normalization, higher-resolution detection, and anchor boxes, as illustrated in detail in Table 2. To enable effective downsampling, YOLO-v2 utilizes a combination of pooling operations and 1 × 1 convolutional layers within its network structure.

YOLO-v2 removes fully connected layers, enabling the model to handle input resolutions from 320 × 320 to 608 × 608, improving detection across multiple scales. This change boosted flexibility and resulted in a 4% mAP improvement, aided by a higher-resolution classifier. Unlike YOLO-v1, YOLO-v2 was trained on 448 × 448 images and fine-tuned for improved accuracy on high-resolution inputs. Batch normalization was introduced to enhance training stability, resulting in an additional 2% improvement in mAP. YOLO-v2 also modified the bounding box prediction, estimating coordinates relative to grid cells rather than absolute positions, resulting in a 5% increase in mAP and improved consistency in predicted box shapes.

**Figure 5:**
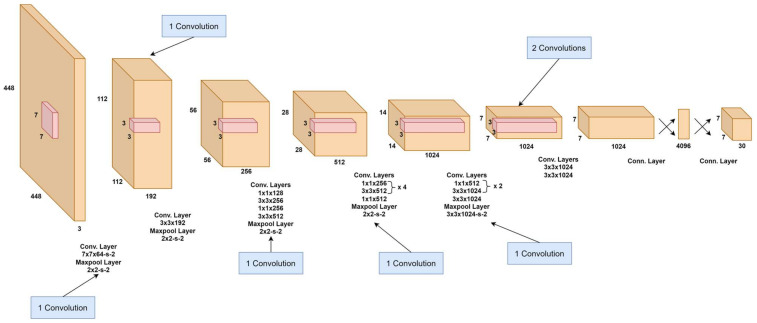
The architecture of YOLO-v2.

YOLO-v2 introduces significant improvements over YOLO-v1, featuring advanced data augmentation, optimized methods, and architectural changes, including the replacement of fully connected layers with convolutional layers and the addition of anchor boxes for enhanced localization. As illustrated in Figure 5, these changes reduce architectural complexity, enhance detection speed, and improve the handling of high-resolution images. The Darknet-19 backbone, with 19 convolutional layers and five max-pooling layers, forms the core of YOLO-v2. The model’s recall rate increased by 7%, although mAP dropped slightly by 0.3%. A K-means clustering approach refines anchor box selection, improving prediction accuracy. Additionally, skip connections inspired by ResNet enhance the model’s ability to detect small objects by merging high- and low-resolution features, contributing an additional 1% to mean Average Precision.

**Figure 6:**
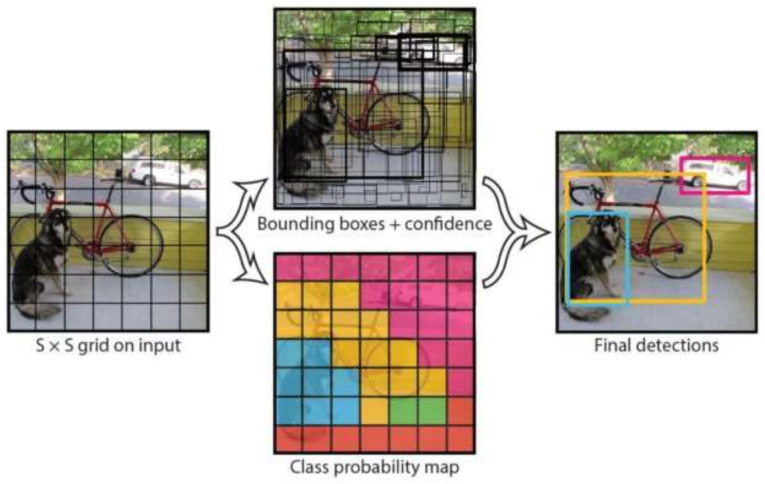
The YOLO model divides the image into an S × S grid. Image source [66].

This design strikes a balance between accuracy and efficiency, making YOLO-v2 lightweight for real-time applications. One significant improvement is the use of anchor boxes, which allows the model to predict bounding boxes more accurately by pre-defining multiple shapes and sizes for objects. This enables YOLO-v2 to handle varying object scales better, unlike YOLO-v1, which struggled with smaller objects, as illustrated in Figure 6. The process of YOLO-v2 involves dividing the input image into an (s×s) grid, where each grid cell predicts bounding boxes and class probabilities. For each bounding box, the network predicts five parameters: the center coordinates (x,y) width, height, and object confidence score. YOLO-v2 uses dimension priority through anchor boxes clustered from training data to enhance localization accuracy. During training, the model enhances object confidence scores and class probabilities by integrating localization loss for box coordinates with classification loss for object categories. Batch normalization across all convolutional layers helps enhance convergence and prevent overfitting. YOLO-v2 also supports multi-scale training, where the input size is periodically changed during training, making the model robust to varying resolutions. As a result, YOLO-v2 delivers a good tradeoff between accuracy and speed, making it suitable for real-time applications such as autonomous driving, surveillance, and robotics.

#### 2.2.3. YOLO-v3

YOLO-v3, introduced by Redmon and Farhadi in 2018 [59], further improves the YOLO framework by enhancing both detection performance and accuracy for small objects while maintaining real-time speed. YOLO-v3 is designed for object detection and multi-scale detection tasks. It introduces objectless scores in bounding box predictions and connects the backbone network layers to predictions at three different granularities, enhancing the detection of smaller objects. The architecture builds upon the success of previous versions, introducing several new ideas.

**Figure 7:**
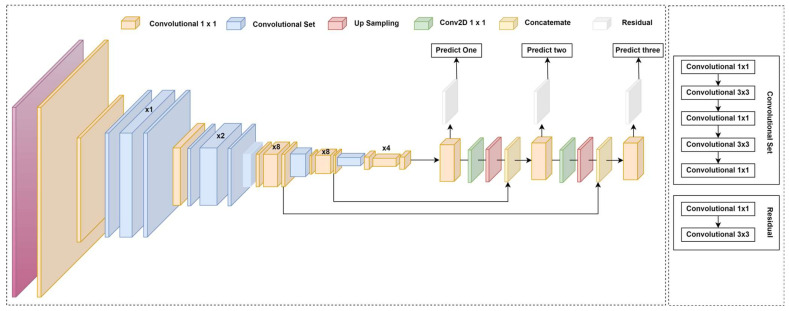
The architecture of YOLO-v3.

It utilizes a more advanced feature extractor called Darknet-53, which consists of 53 convolutional layers. It utilizes residual connections, similar to ResNet, to enhance gradient flow and improve model stability, as illustrated in Figure 7. This version also adopts a Feature Pyramid Network (FPN) structure, allowing YOLO-v3 to detect objects at multiple scales by predicting three layers. The process flow of YOLO-v3 architecture involves various key components, including 1 × 1 convolutional layers, convolutional sets, up-sampling layers, and concatenation operations, which enhance feature extraction and fusion across scales. The network employs residual connections to facilitate better gradient propagation and stability. Conv2D 1 × 1 layers are used for efficient feature processing before final predictions. The integration of these components enables YOLO-v3 to capture rich spatial information while maintaining computational efficiency.

Furthermore, YOLO-v3 diverges from using SoftMax for multi-class predictions, instead assigning independent logistic classifiers for each class, making it more suitable for datasets with overlapping labels. Although YOLO-v3 achieves higher accuracy than its predecessors, it is more computationally intensive and requires more resources.

Instead of using the SoftMax function for classification, the model adopted binary cross-entropy loss, enabling the assignment of multiple labels to a single bounding box. The architecture also introduced a deeper feature extraction network called Darknet-53, which incorporates 53 convolutional layers with residual connections. This design replaced traditional max-pooling layers with convolutional layers that utilize strides, emphasizing residual learning for improved gradient flow. Additionally, this model transitioned from evaluating the PASCAL VOC dataset [67] to utilizing the Microsoft COCO dataset [68], which has since become the standard benchmark for subsequent YOLO versions.

To improve anchor box selection, K-means clustering [69] was employed to create eight anchor boxes across three different feature map scales. Smaller anchor boxes were assigned to higher-resolution feature maps, enhancing localization precision. Another architectural enhancement was the integration of a Spatial Pyramid Pooling (SPP) module within the backbone network, which helped broaden the receptive field and capture multi-scale contextual information.

YOLOv3, whose architecture is detailed in Table 3, performs object detection across three different scales, 13 × 13, 26 × 26, and 52 × 52, based on an input image size of 416 × 416. These multi-scale predictions are facilitated by a deep residual network structure, where each detection scale is enriched with feature maps learned through multiple convolutional and residual blocks. Each grid cell at these scales is associated with three anchor boxes, enhancing the model’s ability to detect objects of varying sizes. These architectural enhancements contribute to a 2.7% improvement in AP-50. Overall, YOLOv3 demonstrates competitive performance, achieving an Average Precision (AP) of 36.2% and an AP-50 of 60.6% while maintaining a real-time inference speed of 20 frames per second (FPS), outperforming several prior high-performing object detection models.

#### 2.2.4. YOLO-v4

**Figure 8:**
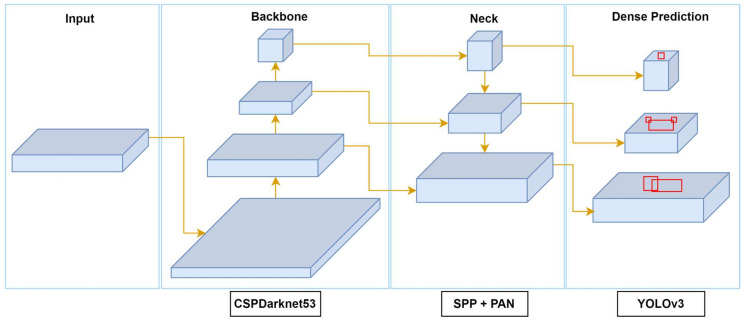
The architecture of YOLO-v4.

YOLO-v4, released in 2020 by Bochkovskiy [60], represents a significant advancement in object detection by enhancing both accuracy and speed, making it well suited for real-time applications on consumer hardware. It is designed for object detection and multi-scale detection tasks, with improvements aimed at better detecting smaller objects and optimizing performance for diverse object sizes, as illustrated in Figure 8. The backbone of YOLO-v4 is CSPDarknet-53, an evolution of Darknet-53 that incorporates cross-stage partial (CSP) connections to enhance gradient flow and reduce computational costs. YOLO-v4 also introduces the use of Spatial Pyramid Pooling (SPP) layers to increase the receptive field by aggregating features at multiple scales and Path Aggregation Networks (PANets) to enhance the flow of low-level and high-level features, improving the detection of objects of varying sizes.

To optimize the speed–accuracy tradeoff, YOLO-v4 integrates several “Bag of Specials” techniques, such as Mish activation, and “Bag of Freebies” methods, like data augmentation, Complete IoU (CIoU) loss, and mosaic augmentation. These techniques collectively enhance training without negatively impacting inference speed. Mosaic augmentation, for instance, exposes the model to diverse contexts by randomly combining four images into one, which improves generalization. The detection pipeline in YOLO-v4 follows the fundamental structure of previous YOLO versions, with key enhancements at multiple stages. The input image is divided into a grid, and predictions are made at three different scales to handle objects of various sizes.

Each grid cell predicts multiple bounding boxes using anchor boxes, with parameters for coordinates (x,y), width, height, and object confidence scores. During training, YOLO-v4 employs multi-scale training, which dynamically adjusts the input resolution to enhance robustness across different image sizes. The loss function utilizes CIoU, which incorporates both overlap and aspect ratio considerations, thereby improving localization precision. These innovations enable YOLO-v4 to achieve state-of-the-art accuracy while maintaining high inference speeds. Its efficient detection pipeline and robust performance make it ideal for real-time applications such as autonomous driving and video surveillance systems.

The YOLO-v4 architecture is designed to strike an effective balance between detection accuracy and computational efficiency, as outlined in Table 4. The input image, typically resized to 608 × 608 pixels, is first normalized before passing through the network. The architecture begins with a 3 × 3 convolutional layer featuring 32 filters, followed by a 3 × 3 convolution with 64 filters and a stride of 2 for downsampling, which facilitates the extraction of low-level features. To enhance learning efficiency and reduce computational overhead, YOLOv4 integrates CSP blocks at various stages of the network. These blocks split the feature maps into two paths—one processed through multiple convolutional layers and the other bypassing them—before merging, thereby improving gradient flow and reducing redundant computations. As the network deepens, successive CSP blocks are applied at increasing depths (64, 128, 256, and 512 filters), culminating in an SPP module that aggregates contextual features at multiple receptive fields. Finally, the YOLO head performs multi-scale detection at three output resolutions, enabling robust object detection across a range of object sizes.

#### 2.2.5. YOLO-v5

**Figure 9:**
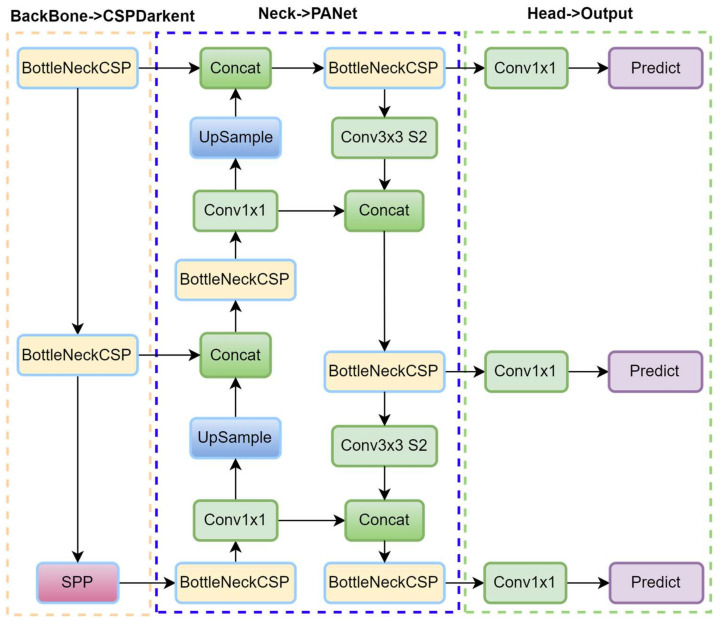
The architecture of YOLO-v5.

Released by the Ultralytics team in 2020 [72], YOLO-v5 signifies a significant advancement in object detection, combining speed, accuracy, and simplicity to meet the needs of real-time applications. Designed for object detection and basic instance segmentation tasks, YOLO-v5, as illustrated in Figure 9, incorporates several enhancements in training techniques and network design, enabling it to perform efficiently even on consumer-grade hardware. It reduces network parameters, utilizing a Cross-Stage Partial Network (CSPNet) for the head and PANet for the neck, and it incorporates a residual structure and auto-anchor functionality, resulting in faster and lighter performance compared to previous YOLO versions. Additionally, it utilizes Mosaic augmentation, which randomly combines four images during training to expose the model to varied contexts, thereby improving generalization. These enhancements make YOLO-v5 extremely easy to train and suitable for inference on individual images, batch images, video feeds, and webcam ports, with excellent transferability and reusability of weights.

The YOLO-v5 process follows a streamlined structure. The input image is first resized to 640 × 640 pixels and normalized. It is then passed through the CSPDarknet backbone, where features are extracted at multiple scales. These features are fed into the neck, typically a combination of Feature Pyramid Network (FPN) and Path Aggregation Network (PANet) layers, ensuring that both high-level and low-level features are fused effectively to detect objects of varying sizes. The detection head predicts bounding boxes, object confidence scores, and class probabilities. Predictions are made at three different scales, making YOLO-v5 robust for detecting both large and small objects. YOLO-v5 applies non-maximum suppression (NMS) to eliminate overlapping boxes and retain the most confident predictions during inference. The model’s loss function incorporates Complete IoU (CIoU) loss, which considers the overlap, distance, and aspect ratio between predicted and ground-truth boxes, improving localization precision. Multi-scale training further enhances robustness by dynamically adjusting the input resolution during training.

With these optimizations, YOLO-v5 achieves a substantial trade-off between speed and accuracy, making it well suited for applications such as autonomous driving, surveillance, and industrial inspection. YOLO-v5’s combination of innovative training techniques and lightweight architecture allows it to deliver real-time performance with minimal computational resources, ensuring broad applicability across diverse domains. Table 5 presents a comparative analysis of the five variants of YOLO-v5 (n, s, m, l, and x), highlighting the tradeoffs between model size, accuracy, and computational efficiency.

#### 2.2.6. YOLO-v6

**Figure 10:**
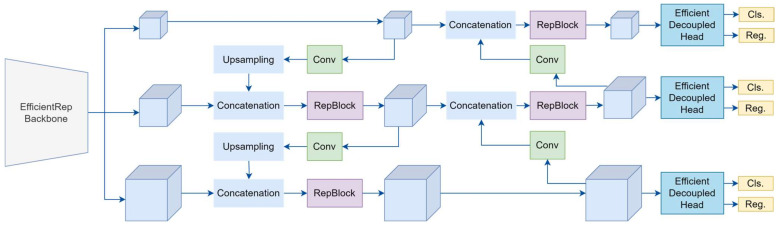
The architecture of YOLO-v6.

YOLO-v6, developed by the Meituan Vision AI Department team [61], is built upon the YOLO framework. It focuses on achieving a superior balance between speed and accuracy, making it suitable for industrial and real-time applications. Designed for object detection and instance segmentation tasks, YOLO-v6 introduces several architectural upgrades, including a redesigned EfficientRep Backbone and Rep-PAN Neck, with a decoupled network head that separates different features from the final head, as illustrated in Figure 10. This configuration improves the model’s detection of small objects and enables anchor-free training, though it is somewhat less stable and flexible compared to YOLO-v5. The architecture also includes a Feature Pyramid Network (FPN) with Bi-directional Cross-Stage Partial (BiCSP) layers and an optimized detection head, enhancing feature extraction and fusion while maintaining computational efficiency.

YOLO-v6 also integrates Skew Intersection over Union (SIoU) loss for improved localization precision, enhancing the model’s accuracy in object detection. The process flow of YOLO-v6 begins with input pre-processing, where images are resized to 640 × 640 pixels and normalized. These images pass through the EfficientRep backbone, which combines residual structures with re-parameterized convolutions, ensuring efficient feature extraction while reducing inference time. The backbone outputs multi-scale feature maps, which are fed into the neck and consist of FPN and BiCSP layers. These layers aggregate features across scales, enabling the robust detection of objects of varying sizes.

The detection head then processes the features, predicting bounding boxes, class probabilities, and object confidence scores across multiple scales. During training, the SIoU loss enhances the alignment between predicted and ground-truth boxes, while multi-scale data augmentation improves the model’s generalization. During inference, the model applies non-maximum suppression (NMS) to filter out redundant bounding boxes, retaining only the most confident predictions. YOLO-v6’s modular design and efficient computation make it well suited for real-time applications, such as video surveillance, autonomous systems, and manufacturing.

The streamlined architecture offers enhanced speed and accuracy over its predecessors while remaining flexible for deployment on devices with varying computational resources. Table 6 presents a comparative analysis of the four variants of YOLO-v6 (n, s, m, and l), highlighting the tradeoffs between model size, accuracy, and computational efficiency.

#### 2.2.7. YOLO-v7

YOLO-v7, developed by Chien-Yao et al. [62], sets a new benchmark in real-time object detection by introducing innovative methods to enhance speed and accuracy without increasing inference costs. Designed for tasks such as object detection, object tracking, and instance segmentation, YOLO-v7 enhances its architecture by incorporating layer aggregation through E-ELAN, optimizing network design, and utilizing trainable “Bag-of-Freebies” methods that improve model performance during training without compromising real-time inference. These innovations, combined with a 35% reduction in network parameters, increase both speed and accuracy, making training and inference more efficient. The model also supports scaling concatenation-based structures, balancing computational demands with detection precision. Among its advancements, YOLO-v7 introduces optimized feature re-parameterization modules and dynamic label assignment, which assigns detection tasks to specific output layers to improve computational efficiency and detection accuracy. The model features two new scaling methods, “extend” and “compound scaling”, allowing model performance to be optimized across various parameters and tuned for different hardware configurations. The architecture uses this extended design to enhance detection accuracy while optimizing computation and parameter usage.

Following preprocessing, input images are resized to 640 × 640 pixels. YOLO-v7 passes data through a re-parameterized backbone network that enhances feature extraction through combinations of modular convolutional and linear operations. The detection head processes the multi-scale feature maps and outputs bounding boxes, class probabilities, and confidence scores. During inference, non-maximum suppression (NMS) removes redundant bounding boxes, providing only the most accurate predictions. YOLO-v7’s innovative contributions set new standards for real-time object detection and inference speed, making it suitable for applications such as video analysis, robotics, and embedded systems. Its streamlined, scalable architecture ensures efficient processing while maintaining high task accuracy. Table 7 presents a comparative study of the five variants of YOLO-v7 (Tiny, v7, X, E6, and D6), highlighting the trade-offs between model size, accuracy, and computational efficiency.

#### 2.2.8. YOLO-v8

YOLO-v8 [76] represents the latest iteration in the YOLO series, pushing the boundaries of real-time object detection by integrating improved model components and novel processing techniques that enhance precision and computational efficiency. YOLO-v8 is designed for object detection, instance segmentation, classification, oriented detection, and pose/keypoint detection. It features a modular architecture with anchor-free detection, decoupled localization, classification heads, an adaptive anchor-free methodology, and improved non-maximum suppression (NMS). These advancements enhance accuracy across various image sizes, enable flexible scaling, offer high adaptability in real-time applications, and reduce false positive rates. Developed with a focus on adaptability, YOLO-v8 introduces modular elements that enable users to tailor the model’s depth and width according to hardware constraints and application-specific needs. Significant advancements in YOLO-v8 include improved feature extraction techniques and better separation of the detection head, which manages localization and classification tasks independently to enhance both accuracy and speed.

Additionally, YOLO-v8 utilizes adaptive anchor-free detection and an enhanced non-maximum suppression (NMS) technique, thereby reducing false positives and improving the clarity of output predictions. This architecture also emphasizes model scalability by supporting various image sizes while maintaining high accuracy in computationally constrained environments. With these innovations, YOLO-v8 sets new benchmarks for performance in diverse applications, including autonomous systems, surveillance, and augmented reality, offering substantial flexibility and reliability for modern real-time object detection tasks. Table 8 presents a comparative study of the five variants of YOLO-v8 (n, s, m, l, and x), highlighting the trade-offs between model size, accuracy, and computational efficiency.

#### 2.2.9. YOLO-v9

YOLO-v9, developed by Wang et al. [63], represents a significant advancement in the YOLO series, marking a substantial leap in detection capabilities through the integration of Transformer-based attention mechanisms directly into its backbone. This enhancement distinguishes it from earlier versions by enabling YOLO-v9 to navigate complex scenes more effectively. The self-attention mechanism within transformers captures global contextual relationships, allowing the model to differentiate overlapping defects, such as intertwined threads or adjacent tears, which are often challenging for traditional CNN-based models. By analyzing the entire spatial context, YOLO-v9 ensures accurate localization and classification of defects, even in densely packed or cluttered environments.

YOLO-v9 focuses on object detection and instance segmentation, featuring substantial enhancements such as optimized tokenization for more efficient feature representation and a unified module for object tracking and detection. These upgrades result in faster processing of complex scenes, enhanced object-tracking performance, higher accuracy in multi-object environments, and improved efficiency with high-resolution images. Furthermore, the optimized tokenization process within the Transformer layers enhances feature representation while reducing computational load. A noteworthy feature is a unified module for object tracking and detection. It equips the model with real-time tracking capabilities, making it well suited for dynamic applications such as autonomous driving and surveillance systems.

Additionally, YOLO-v9’s architecture facilitates seamless adaptation to high-resolution images, ensuring precise object detection across various image sizes without significantly increasing computational demands. By leveraging these innovations, YOLO-v9 delivers high-speed, accurate detection, setting new standards for applications requiring real-time tracking and recognition of multiple objects in diverse contexts. Table 9 presents a comparative study of the five variants of YOLO-v9 (t, s, m, c, and e), highlighting the trade-offs between model size, accuracy, and computational efficiency.

#### 2.2.10. YOLO-v10

**Figure 11:**
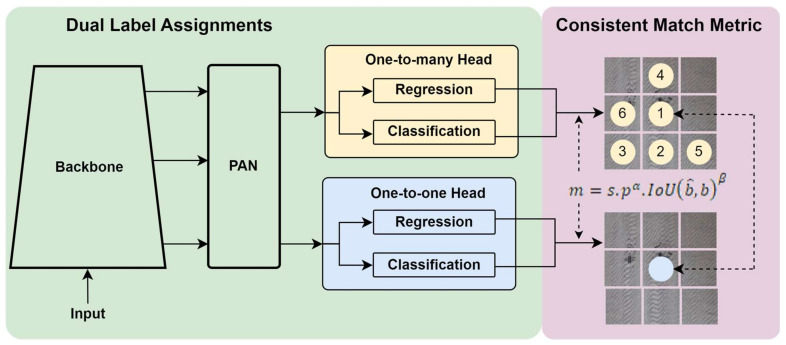
The architecture of YOLO-v10.

YOLO-v10, developed by Wang et al. [64], builds on the advancements of its predecessors by introducing a hybrid attention mechanism that combines Transformer and convolutional attention layers within its architecture. As shown in Figure 11, this dual-attention approach allows YOLO-v10 to process intricate spatial and contextual details with improved precision, particularly in dense or cluttered scenes. Unlike YOLO-v9, which solely relied on Transformer-based attention in the backbone, YOLO-v10 strategically integrates convolutional attention in earlier layers to efficiently capture local features. In contrast, the Transformer layers focus on the global context.

This combination is especially effective for overlapping defect detection, as convolutional attention focuses on fine-grained details, such as subtle edges or textures. In contrast, Transformer-based attention analyzes the broader spatial relationships, ensuring accurate localization and classification of overlapping defects. The YOLO-v10 architecture adopts a Dual Label Assignment strategy, comprising a one-to-many head and a one-to-one head to enhance detection performance. The pipeline begins with an input module and a backbone network for hierarchical feature extraction. A Path Aggregation Network (PAN) then processes these extracted features to refine multi-scale representations. YOLO-v10 utilizes a one-to-many head that is responsible for regression and classification tasks, utilizing multiple label assignments to enhance recall and robustness. Simultaneously, a one-to-one head performs regression and classification using a strict one-to-one matching approach, ensuring precise localization and minimizing duplicate detections. The final Consistent Match Metric further enhances detection stability by refining the label assignment process, improving object tracking and classification consistency.

Tasks for YOLO-v10 involve object detection, featuring significant enhancements that combine hybrid convolutional and Transformer structures for improved spatial and contextual feature extraction. These improvements are designed explicitly for low-resource environments, enabling YOLO-v10 to excel in applications with limited computational resources. Additionally, YOLO-v10 incorporates advanced lightweight modules for on-device deployment, significantly improving edge-device performance, adaptability to low-power hardware, and maintaining high accuracy and speed on resource-constrained devices. YOLO-v10 further refines tokenization and feature encoding processes, optimizing memory usage and computational efficiency. Another breakthrough in YOLO-v10 is its adaptive resolution scaling feature, which dynamically adjusts the processing resolution based on object density and scene complexity. This ensures that YOLO-v10 maintains high detection performance across various image sizes and resolutions without sacrificing speed or accuracy. Moreover, YOLO-v10 introduces an enhanced unified detection and tracking module, offering advanced tracking capabilities that are particularly beneficial for applications in autonomous robotics, intelligent surveillance, and industrial automation.

With experiments conducted on the COCO dataset [79], YOLO-v10 continues to push the boundaries of real-time multi-object detection by maintaining the YOLO tradition of high speed and accuracy. It enables applications requiring both high precision and contextual awareness across diverse real-world scenarios. Table 10 compares six variants of YOLO-v10 (n, s, m, b, l, and x). Overall, YOLO-v10 outperforms its predecessors and other state-of-the-art models in accuracy and efficiency. For instance, YOLO-v10-s is 1.8 times faster than YOLO-v10-m while maintaining a competitive Average Precision (mAP). Similarly, YOLO-v10-b achieves 46% lower latency and 25% fewer parameters than YOLO-v10-l while maintaining comparable performance levels.

#### 2.2.11. YOLO-v11

YOLO-v11, developed by the Ultralytics team [72], refines YOLO with advanced adaptive learning and intelligent feature distillation for improved detection efficiency. It builds on YOLO-v10’s dual-attention system, incorporating a multi-scale attention architecture that dynamically adapts its focus across network stages, thereby enhancing the detection of both small and large objects. This enables YOLO-v11 to detect both acceptable defects, such as micro-tears, and more significant structural issues within complex scenes, including variations in fabric texture. The model utilizes spatial attention to refine feature maps at various resolutions, thereby enhancing detection performance under diverse lighting conditions.

YOLO-v11 incorporates dynamic model scaling, enabling the architecture to adjust its depth and width based on available hardware resources, thereby ensuring efficient performance on both powerful GPUs and edge devices. Additionally, semi-supervised learning leverages labeled and unlabeled data, reducing the need for extensive manual annotations. Its context-aware, self-supervised fine-tuning enhances adaptation to real-world fabric conditions, such as variations in lighting and texture.

**Figure 12:**
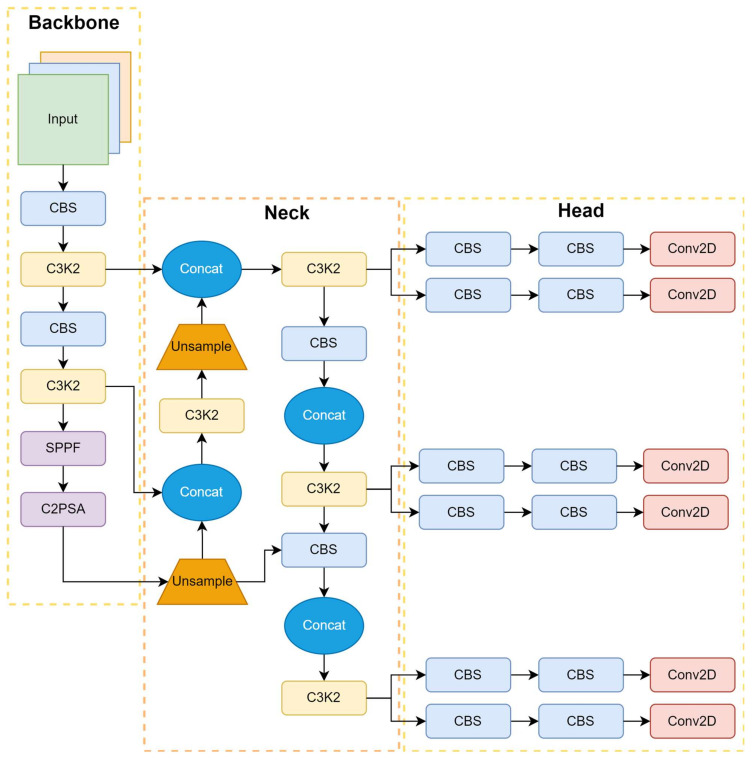
The architecture of YOLO-v11.

The model also introduces an optimized, lightweight detection head and adaptive non-maximum suppression (NMS), which improves computational efficiency and reduces false positives. YOLO-v11’s architecture, as shown in Figure 12, featuring a backbone, neck, and head, ensures efficient feature extraction, spatial refinement, and detection output, making it a robust solution for real-time fabric defect detection in industrial environments.

This architecture retains YOLO’s trademark speed while delivering improved accuracy across applications that require rapid and precise detections, such as real-time sports analysis, autonomous vehicle perception, and complex industrial automation systems. By providing detailed insights into these mechanisms, YOLO-v11 sets a new benchmark in automated quality control for the textile industry. Table 11 compares five YOLO-v11 variants: YOLO-v11 (n, s, m, l, and x). It highlights the trade-offs among model size, accuracy, and computational efficiency. All models from YOLOv5 to YOLOv11 operate on input images of 640 × 640 pixels, with YOLOv7 also supporting 1280 × 1280 pixels. Their performance varies across key metrics such as mean Average Precision (mAP) at different thresholds (mAP 50 and mAP 50-95), CPU ONNX inference time (ms), Speed A100 TensorRT (ms), number of parameters (M), floating-point operations per second (FLOPs), and FPS. These performance metrics were obtained from experiments on pre-trained detection models using the COCO dataset.

### 2.3. Comparative Analysis of YOLO Variants

The comparative analysis of YOLO variants reveals key advancements that have a significant impact on fabric defect detection. Earlier models, such as YOLO-v3 and YOLO-v4, introduced multi-scale detection and improved anchor box clustering, enhancing their ability to localize defects of varying sizes. However, these models still struggled with fine-grained texture variations and overlapping defects. YOLO-v5 and YOLO-v7 improved upon these limitations by introducing better feature fusion mechanisms and lighter architectures, thereby balancing speed and accuracy, making them highly suitable for real-time manufacturing environments. More recent models, such as YOLO-v9, YOLO-v10, and YOLO-v11, leverage Transformer-based attention mechanisms and adaptive learning, allowing them to capture intricate fabric textures and detect subtle micro-defects more effectively. While these models achieve higher accuracy, they come with increased computational demands, making them more suited for high-end GPU environments rather than resource-limited edge devices. Ultimately, the choice of YOLO variant depends on the trade-off between accuracy, speed, and hardware availability, with YOLO-v5 and YOLO-v7 remaining strong choices for real-time applications, while YOLO-v10 and YOLO-v11 excel in high-precision defect detection for quality control in advanced manufacturing. Table 12 compares the strengths and weaknesses of various YOLO variants, illustrating the trade-offs between speed, accuracy, and complexity as these architectural components evolve to meet the demands of real-world object detection tasks.

### 2.4. The Evolution of Loss Function on YOLO Variants

The evolution of the YOLO framework, from YOLO-v1 to YOLO-v11, has demonstrated significant advancements in improving real-time object detection [82]. A key focus of this evolution has been refining loss functions to enhance accuracy while maintaining computational efficiency [83]. In object detection, loss functions play a crucial role in determining how effectively a model learns to localize and classify objects accurately, as well as refine bounding boxes. YOLO works by dividing an image into a grid and predicting bounding boxes along with class probabilities, with loss functions guiding the optimization of these predictions during training [84]. The impact of loss functions on fabric defect detection is noteworthy. Each YOLO variant introduces innovations in loss functions to tackle the challenges of detecting small, irregular, or overlapping fabric defects, as shown in Table 13. The early YOLO versions from YOLO-v1 to YOLO-v2 employed Mean Squared Error (MSE) and Sum Squared Error for bounding box regression [85]. However, these loss functions struggled with scale variance and aspect ratio mismatches, leading to poor localization of minor fabric defects.

With YOLO-v3, the introduction of Generalized IoU (GIoU) and Distance IoU (DIoU) improved localization accuracy by penalizing bounding boxes based on their shape and overlap, making it easier to detect subtle fabric inconsistencies [86]. YOLO-v4 and YOLO-v5 further enhanced localization with Complete IoU (CIoU), which considers both distance and aspect ratio, ensuring more precise defect boundary detection, especially in high-resolution textile images [87]. Comparative studies indicate that CIoU outperforms DIoU in fabric defect detection due to its better alignment with the geometric characteristics of textile defects, particularly in high-resolution images where localization precision is paramount. Early models primarily relied on binary cross-entropy (BCE) for classification, which was sufficient for basic defect classification. However, BCE struggled with imbalanced datasets. YOLO-v4 and YOLO-v5 integrated Focal Loss to mitigate class imbalance by reducing the influence of easily classified defects while emphasizing harder-to-detect anomalies, such as faint discolorations and micro-tears in fabrics [88].

YOLO-v6 and later models integrated Distribution Focal Loss (DFL) and VariFocal Loss, further refining probability estimation and improving small object detection [89]. These models effectively distinguish minor fabric defects, such as micro-tears or subtle stains. YOLO-v9 adopted L1 Loss to enhance convergence speed, making it particularly effective for detecting fine-textured fabric defects in real-time edge-based systems, where computational efficiency is crucial [90]. The latest models, YOLO-v10 and YOLO-v11, employ hybrid loss functions that incorporate Coordinate Loss, Confidence Loss, and CloU (Contextual IoU). These refinements enhanced detection in dense or complex textile patterns, resulting in higher accuracy in distinguishing overlapping defects. By leveraging these advanced loss functions, newer YOLO models have enhanced the ability to detect small and complex fabric defects more effectively, ensuring that defect localization remains accurate even in high-speed manufacturing environments. These improvements bridge the gap between academic advancements and industrial applications, making real-time defect detection more reliable and efficient for modern textile quality control systems.

### 2.5. Improvements in YOLO Versions and Application in Fabric Defect Detection

The YOLO family of models has undergone significant advancements since its inception, with each version introducing features that address specific challenges in object detection. Among these advancements, Transformer-based attention mechanisms, multi-scale detection [91], and adaptive learning have emerged as critical techniques, enhancing detection capabilities across various YOLO versions. Transformer-based attention mechanisms, first introduced in YOLO-v9 [63] and refined in YOLO-v10 [64], leveraged the self-attention capability of transformers to capture long-range dependencies and contextual relationships between objects within an image. This approach is particularly advantageous in complex scenes where overlapping objects, diverse textures, or cluttered backgrounds are common place [92].

For example, in fabric defect detection, Transformers can differentiate between genuine defects and overlapping patterns by analyzing global spatial relationships [93]. YOLO-v9 integrates Transformer-based attention directly into its backbone, enabling the model to process global contextual information effectively and improve its ability to detect minor defects that larger patterns might obscure. Building on this, YOLO-v10 combines Transformer-based global attention with convolutional local attention, creating a hybrid attention system [94] that ensures precise micro-defect localization while maintaining a broader contextual understanding. This combination is highly effective in scenarios such as textile manufacturing, where both macro- and micro-level defect detection are critical.

Multi-scale detection, introduced in YOLO-v3 [65] and refined in subsequent versions, allows models to detect objects of varying sizes by analyzing feature maps at multiple resolutions. This technique is crucial for applications like fabric defect detection, where defects range from small pinholes to large tears. YOLO-v3 utilizes a Feature Pyramid Network (FPN) to predict objects at three distinct scales, thereby significantly enhancing its ability to detect small and overlapping objects. YOLO-v4 and YOLO-v5 further enhance this by integrating advanced neck architectures, such as Path Aggregation Networks (PANets) [95], which improve the fusion of low-level and high-level features. In YOLO-v7 and later versions, multi-scale detection is complemented by anchor-free mechanisms, streamlining the process and reducing computational overhead [96]. This is particularly beneficial for real-time applications, as it maintains high detection accuracy while improving inference speed.

Prominently featured in YOLO-v11, adaptive learning techniques include dynamic model scaling [97], semi-supervised learning [98], and context-aware self-supervised fine-tuning [99]. These methods enhance the model’s generalization and robustness, enabling it to perform well across diverse datasets and real-world scenarios. Dynamic model scaling adjusts the depth and width of the network based on computational resources and scene complexity, ensuring efficient operation on both high-performance GPUs and resource-constrained edge devices [100]. Semi-supervised learning leverages unlabeled data alongside labeled datasets to enhance detection capabilities in underrepresented scenarios [101], which is particularly beneficial for fabric defect detection, where obtaining labeled datasets can be both expensive and time-consuming [102]. Context-aware self-supervised fine-tuning leverages the inherent structure of input data to refine feature representations without explicit labels, improving detection accuracy in complex environments, such as those with varied lighting or intricate fabric patterns [103].

These advancements demonstrate the versatility and adaptability of YOLO models in addressing the unique challenges of fabric defect detection [104]. A consolidated summary of these advancements, including the YOLO variant and its improvements, along with their results on fabric defect detection, is provided in Table 14. To address the issue of dataset diversity, future research should focus on developing synthetic datasets using generative adversarial networks (GANs) to simulate various fabric textures and defect types [105]. Additionally, lightweight YOLO models optimized for edge devices could enhance real-time performance in resource-constrained environments [106]. By leveraging Transformer-based attention [107], multi-scale detection [108], and adaptive learning [109], YOLO models continue to set benchmarks in automated quality control and real-time object detection [110].

The reviewed advancements in fabric defect detection highlight common trends and ongoing challenges. Attention mechanisms, anchor clustering, and multi-scale feature extraction have significantly enhanced detection accuracy, particularly for minor defects such as micro-tears and uneven dyeing. Among the YOLO variants, YOLO-v5 and YOLO-v7 have demonstrated an optimal balance between real-time performance and precision, making them well suited for high-speed manufacturing environments. Table 15 further illustrates the evolution of YOLO models, highlighting key architectural enhancements that have significantly improved detection capabilities. For instance, YOLO-v5 integrates CSP-Darknet53 with a Spatial Pyramid Pooling-Fast (SPPF) module and an anchor-free split Ultralytics head, optimizing the tradeoff between accuracy and speed. YOLO-v7, which leverages model reparameterization and dynamic label assignment, further improves efficiency. Recent versions, such as YOLO-v10 and YOLO-v11, incorporate self-attention mechanisms to capture long-range dependencies, depth-wise separable convolutions to optimize computational efficiency, and Transformer-based feature fusion for enhanced contextual understanding in complex detection scenarios. Despite advancements, fabric defect detection remains challenging due to texture diversity, lighting variations, and overlapping defects. Further model optimization and domain-specific improvements are needed.

## 3. Fabric Defect Detection Applications, Methods, and Datasets

This section provides a comprehensive review of applications that utilize various YOLO models to identify and localize fabric defects, spanning traditional manufacturing to modern automated quality control systems. In practical industrial environments, fabric defect detection systems powered by YOLO-based models are adopted across various stages of textile manufacturing. For instance, automated weaving and knitting lines enable real-time inspection of fabric surfaces to detect flaws such as holes, stains, and misweaves, thereby reducing material waste and enhancing production efficiency.

In the dyeing and printing processes, YOLO-integrated vision systems help identify color inconsistencies and printing misalignments, ensuring that stringent quality standards are upheld. Furthermore, smart textile factories have incorporated YOLO-driven models into Industry 4.0 frameworks, integrating defect detection with real-time monitoring dashboards and production control systems. In fabric grading and sorting units, YOLO-based detection results are utilized to categorize fabrics into quality categories based on defect severity, thereby facilitating automated quality assurance and informed decision-making. Additionally, specialized applications, such as fiberglass fabric inspection and impurity detection in the cotton processing industry, exemplify how YOLO models can be customized for domain-specific detection tasks. These practical implementations highlight the transformative impact of YOLO-based systems in modernizing textile inspection workflows, enabling scalable, high-speed, and reliable quality control in industrial settings.

**Figure 13:**
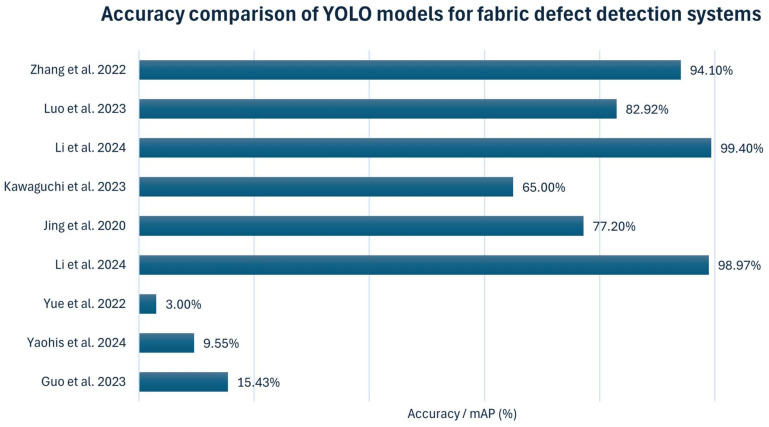
Accuracy comparison of YOLO models for fabric defect detection systems [46,87,111,112,113,114,115,116,117].

Table 16 and Figure 13 present the detection accuracy (mAP) of different studies using YOLO-based models for fabric defect detection. We explore the various techniques that enhance YOLO’s performance, particularly in fabric defect detection applications. These include modified feature extraction, integration with Transformer model networks, and optimized anchor boxes for capturing fine-grained details of fabric. Through these innovations, YOLO-based models are transforming the landscape of fabric inspection by enhancing accuracy, speed, and adaptability across diverse industrial settings. Ye et al. [82] enhanced YOLO-v7 with deformable convolution and an attention mechanism, improving mAP by 15.43%, particularly in detecting subtle and overlapping defects, making it well suited for complex fabric textures. Similarly, Yaohis et al. [111] proposed YOLO-v7-tinier as an efficient and compact method for fabric defect detection, introducing several critical improvements to the YOLO-v7-tiny model. These enhancements include partial convolution, a new module called Dilated Spatial Pyramid Pooling Fast Cross Stage Partial Concat, and a convolutional structure with an attention mechanism, improving detection accuracy and speed while reducing the number of parameters. In experiments, YOLO-v7-tinier achieved a 9.55% improvement in mean Average Precision (mAP) and a 10.81% reduction in parameters compared to the original YOLO-v7 model. This approach addresses the model’s low recognition accuracy and poor real-time performance in online fabric defect detection tasks.

Yue et al. [112] proposed an improved YOLO-v4-based target detection algorithm for fabric defect detection, utilizing data augmentation, anchor clustering, new prediction layers, attention mechanisms, and optimized loss functions to detect tiny objects within fabric defects accurately. These techniques collectively enhance the model’s ability to detect small targets and improve its overall performance in fabric defect detection. Comparably, Li et al. [113] proposed an improved YOLO-v4-based algorithm for fabric surface defect detection, incorporating various enhancements such as anchor determination using the Density-Based Spatial Clustering of Applications with Noise (DBSCAN) algorithm to optimize the number of anchors, which are crucial for accurately localizing fabric defects within images. Additional improvements include enhancements to the feature extraction network, fusion detection experiments, and performance verification. These improvements collectively enhance the model’s robustness, feature extraction capabilities, and overall performance in fabric defect detection.

Jing et al. [46] proposed a high-performance, real-time fabric defect detection method based on an improved YOLO-v3 model to enhance defect detection rates and fabric product quality. Critical enhancements include dimension clustering of target frames based on fabric defect sizes and the use of the K-means algorithm to optimize anchor box dimensions and quantities. The model also integrates low-level and high-level features, adding a YOLO detection layer to multi-scale feature maps, which enhances its applicability to defect detection in gray and checked fabrics. The improved model achieves an error detection rate of less than 5% for these fabrics. Experimental results demonstrate that this approach outperforms the standard YOLO-v3 in terms of defect detection accuracy and reduces the error rate. Kawaguchi et al. [114] proposed a real-time fabric defect detection system using YOLO to enhance efficiency in textile processing. Unlike the traditional methods, such as CNNs, which require separate object detection and identification stages, real-time performance on manufacturing lines is limited. By implementing YOLO, this study aims to streamline the defect detection process, directly enabling rapid and accurate inspection within the production workflow. The authors envision that this approach could lead to the automation of textile defect detection, reducing human error, improving accuracy, and lowering labor costs in fabric inspection.

Li et al. [115] introduced a novel three-stage cascaded network model, Mobile-YOLO, for online detection of defects in fiberglass fabric. The tolerance for various defects in the fiberglass fabric production process differs, and acceptable defects only require relevant information to be recorded and printed. In contrast, intolerable defects necessitate an immediate halt of the loom for handling. The proposed model integrates enhancements in MobileNetV3 and YOLO-v8n, along with attention mechanisms, to improve feature representation, model efficiency, and detection accuracy for different types of defects in fiberglass fabric. Rasheed et al. [19] provide a comprehensive overview of computer vision techniques used in fabric defect detection, evaluating their performance criteria and discussing limitations in existing research. The authors discuss the shift toward automated processes for fabric defect detection, aiming to reduce labor costs and enhance inspection quality. It compares manual inspection processes with automated computer vision-based methods, emphasizing the advantages of automation in detecting fabric defects. It also suggests future research directions to advance automated defect detection in the textile industry.

Luo et al. [116] proposed a lightweight detection detector that leverages an attention mechanism to improve detection accuracy and efficiency, given the significant economic losses caused by fabric defects in the textile industry. This paper emphasizes the importance of automated fabric defect detection. It categorizes existing fabric defect detection methods into four main categories: structural analysis, statistical analysis, frequency domain analysis, and model analysis. These categories employ various techniques, including contour feature extraction, similarity estimation, filter-based methods, and texture feature extraction with convolutional neural networks. By incorporating computer vision, deep learning, and attention mechanisms, the proposed model aims to overcome the limitations of traditional manual defect detection methods and enhance production efficiency in the textile industry. Zhang et al. [117] proposed a multi-channel fusion segmentation method and an improved YOLO-v4 classification and recognition method for impurity detection in machine-picked seed cotton. The improved YOLO-v4 model classifies and recognizes impurities in the segmented images. Impurities such as boll shells, stiff petals, cotton branches, and weeds in machine-picked seeds can affect the quality of the cotton and subsequent processing. Among YOLO variants, YOLO-v5 and YOLO-v7 have demonstrated a balance between speed and accuracy, making them ideal for high-speed production lines.

YOLO versions v9 and v10 have recently leveraged transformers to enhance defect localization, particularly in overlapping or complex textures [118]. Transformers utilize self-attention to capture global contextual relationships within an image, enabling the model to distinguish between closely located defects and overlapping textures [119]. For example, in fabric defect detection, overlapping patterns such as intertwined threads or adjacent tears often confuse traditional CNN-based models due to their localized feature extraction [120]. In contrast, transformers assess the entire spatial context, ensuring that each defect is identified and localized. In YOLO-v10, the hybrid attention mechanism combines Transformer-based global attention with convolutional local attention, improving precision. This dual approach allows the model to focus on the intricate details of overlapping defects while retaining a broader understanding of the surrounding fabric texture. As a result, YOLO-v10 demonstrates superior performance in scenarios with high defect density or complex textures, such as patterned or intricately woven fabrics.

### 3.1. Fabric Defect Detection Datasets

This section presents the datasets for fabric defect detection tasks, providing a comprehensive analysis of their characteristics and associated challenges. These datasets play a crucial role in advancing research by providing benchmarks to evaluate the performance of various YOLO models and other detection algorithms. Key attributes, such as image complexity, resolution, the number of defect classes, and dataset size, illustrate the diversity of these datasets and their year of publication. Despite their significance, challenges persist, including managing the high computational demands of processing large-scale datasets and developing efficient algorithms for real-time applications in industrial settings. For example, the Tianchi AI dataset features high-resolution images (513 × 513 and 1536 × 1536), making it suitable for detailed defect analysis, but it is computationally intensive in real-time scenarios. Similarly, the TILDA dataset, which contains 3200 images across five defect classes, is well suited for examining smaller-scale defects.

Other datasets, such as KTH-TIPS-I and KTH-TIPS-II, provide a broader range of resolutions (200 × 200 to 1280 × 960) and support up to 11 classes, offering versatility for various experimental setups. The AITEX Fabric dataset, featuring high-resolution 4096 × 256 images across seven defect classes, is a strong foundation for training models designed for industrial fabric inspection. Additionally, the TIDA 400 dataset, comprising 25,600 images at a resolution of 64 × 64, emphasizes efficiency and scalability, making it suitable for applications with limited computational resources. Finally, the Lusitano dataset stands out with an extensive collection of 36,000 high-resolution images (4096 × 1024) across 35 defect classes, providing unparalleled diversity for training models to handle complex defect scenarios. The ZJU-Leaper dataset, comprising 98,777 images across 19 classes, provides a substantial resource for deep learning experiments. The YDFID-1 dataset, which has 3830 images at a resolution of 512 × 512 × 3 with 19 defect classes, is particularly valuable for color-based defect detection tasks.

The Fabric Stain Dataset, comprising 466 high-resolution images (1920 × 1080 and 1080 × 1920 pixels) across five classes, is designed to detect specific stain-related defects. The HKU Fabric dataset, comprising 250 images at a resolution of 256 × 256 and six classes, provides a compact yet valuable resource for exploring defect detection in smaller datasets. Lastly, the Brodatz Textures dataset, comprising 700 images with resolutions ranging from 60 × 60 to 512 × 512 and nine classes, remains a classic choice for texture-based defect studies. Table 17 summarizes the key attributes of these datasets, including image resolution, the number of classes, and the publication year, highlighting their value in enhancing the scalability and robustness of fabric defect detection models. By leveraging these datasets, researchers can effectively address the complexities of fabric textures, defect types, and industrial requirements, ultimately advancing the field of automated defect detection.

### 3.2. Textile Manufacturing

The applications related to the automated inspection of fabric defect detection in production focus on detecting issues such as holes, stains, tears, and misaligned patterns. Automated inspection reduces manual inspection costs, enhances production line efficiency by quickly identifying defective fabrics, and maintains product quality by flagging defects early in the process, as shown in Table 18.

Ngan et al. [3] reviewed fabric defect detection methods developed in recent years, with a primary focus on automated defect detection in the textile manufacturing industry. Their study highlights how automated inspection enhances efficiency by detecting defects early, reducing costs, and improving fabric quality control. It is a natural progression to strengthen fabric quality while lowering labor costs compared to traditional human inspection methods. Automated inspection methods achieve a high success rate of over 90% compared to 60–70% for human inspection. Mak et al. [132] studied the application of advanced computer image processing techniques for automated defect detection in textile fabrics. They proposed a new defect detection scheme based on Gabor filters and evaluated its performance using a fabric image database that includes various homogeneous fabric samples. The results of the proposed scheme show accurate defect detection with low false alarm rates, demonstrating its effectiveness and robustness. Real-time tests conducted using a prototype automated defect detection system further confirm the approach’s efficiency and reliability. They also discussed the importance of quality control in the garment manufacturing industry and the limitations of manual inspection, including human error, inconsistent performance, and high inspection costs. It emphasizes the need for automated visual inspection systems to reduce manufacturing costs and enable a more reliable, objective, and consistent quality control process.

Jiang et al. [133] address automated fabric inspection during production, focusing on detecting defects such as holes, stains, tears, and misaligned patterns. For this purpose, several advanced algorithms and techniques have been developed in computer vision and image processing. They mention various approaches that can be applied to fabric defect detection, including structure-based, statistical-based, spectral-based, and model-based detection methods. These methods utilize texture analysis, statistical characteristics, mathematical morphology, and spectral approaches to identify and classify defects in fabric images. Automated inspection systems based on these methods can significantly reduce manual inspection costs, enhance production line efficiency by quickly identifying defective fabrics, and maintain product quality by flagging defects early in manufacturing. By implementing these advanced algorithms and techniques, manufacturers can improve the quality control process and ensure that only high-quality fabrics are delivered to customers.

### 3.3. Defect Detection Algorithms

Fabric defect detection relies on various algorithms tailored to identify imperfections in textile materials. Traditional methods often employ handcrafted features in conjunction with classical machine learning techniques, such as support vector machines (SVMs) and k-nearest neighbors (k-NNs) [134,135]. However, recent advancements in computer vision have shifted the focus toward deep learning-based approaches. For instance, CNNs excel at learning intricate patterns directly from images, making them particularly effective for fabric defect detection [136]. Similarly, object detection frameworks like YOLO and Faster R-CNN have demonstrated strong performance in detecting and localizing defects [137]. Transformer-based models have also emerged, offering improved feature extraction capabilities for complex textures.

Each approach has unique advantages depending on the dataset size, defect complexity, and computational resources. Avudaiamaal et al. [138] discuss the significance of roofing materials in various contexts, including urban development, poverty assessment, and post-disaster building evaluations. They employ the YOLO algorithm, a widely used deep learning model for object detection, to identify and classify different roofing materials from aerial imagery. Kailasam et al. [139] highlight the critical role of fabric defect detection in the textile manufacturing industry, emphasizing its importance in quality control. They also discuss techniques like R-CNN and YOLO-v4, which were developed to create more precise and effective systems for detecting fabric defects. Saberironaghi et al. [140] provide a summary and analysis of current research on defect detection using machine learning methods, including deep learning-based approaches for identifying surface defects. Their work covers supervised, semi-supervised, and unsupervised techniques and discusses current research trends in deep learning-based defect detection methods for X-ray images.

#### 3.3.1. Integration of YOLO Models

Integrating YOLO models into fabric defect detection systems involves a comprehensive workflow that encompasses training, fine-tuning, and deployment, tailored to meet the specific demands of industrial applications. The training begins by preparing a well-annotated dataset comprising images of fabric defects, with bounding boxes and labels identifying defects such as tears, stains, and misaligned patterns. Data augmentation techniques, such as rotation, flipping, cropping, and mosaic augmentation, enhance model robustness by exposing it to diverse fabric textures and defect variations [141]. Optimal anchor box dimensions are determined using clustering algorithms, such as K-means, to ensure precise localization of defects [142]. Fine-tuning pre-trained YOLO models, typically initialized with weights from large-scale datasets like COCO, accelerates their adaptation to fabric-specific datasets [143]. This transfer learning approach reduces training time and computational costs while enabling the model to specialize in identifying fabric defects. Techniques that can be applied, such as layer freezing, preserve general features in the backbone network while updating detection layers for specific tasks.

Transformer-based models have emerged as powerful mechanisms for improving the contextual understanding of YOLO models [144]. By integrating self-attention mechanisms, YOLO models can capture long-range dependencies and relationships between features, enabling better differentiation between defects and background textures. Transformer-based enhancements also improve the detection of overlapping defects by simultaneously analyzing global and local contexts. Incorporating these mechanisms into the YOLO architecture enhances its ability to detect subtle and complex fabric defects, particularly in scenarios involving intricate patterns or multiple defect types within the same region. Attention mechanisms, such as spatial and channel attention, further refine feature extraction by emphasizing critical regions of the input image [145]. Spatial attention focuses on areas with a higher probability of defects, while channel attention highlights the most informative feature maps. This dual focus improves detection accuracy for small or subtle defects that might be overlooked. Integrating attention mechanisms into YOLO’s backbone and neck layers ensures that the model prioritizes relevant features, enhancing its robustness across diverse fabric textures and lighting conditions.

Adaptive anchor box strategies optimize the localization of fabric defects by dynamically adjusting anchor box dimensions to match the varying scales and shapes of defects [146]. Unlike static anchor boxes, adaptive strategies employ data-driven approaches, such as iterative clustering or online learning, to dynamically adjust anchor box sizes during training. This ensures better alignment between predicted and ground-truth bounding boxes, particularly for irregularly shaped or slight defects. Adaptive anchor box strategies significantly enhance the precision and recall of YOLO models, making them more effective in industrial applications where defect variability is high. Deployment in real-world environments emphasizes real-time performance by leveraging hardware accelerations, such as GPUs or edge AI devices, for high-speed inference [147]. Furthermore, post-processing steps, including non-maximum suppression, refine outputs by eliminating redundant predictions. By preparing a custom dataset that reflects real-world complexities, such as lighting variations, texture diversity, and overlapping defects, and employing transfer learning to bridge the gap between generic datasets and domain-specific applications, YOLO models achieve high accuracy and scalability in fabric defect detection.

#### 3.3.2. Post-Detection Processing

Fabric defect detection systems by refining model predictions and enabling actionable insights. One essential step involves filtering out false positives to ensure only genuine defects are flagged, reducing unnecessary interventions and maintaining operational efficiency on production lines [147]. Additionally, a severity assessment is performed to categorize detected defects into levels, such as minor or critical, enabling manufacturers to prioritize actions based on defect severity [148]. For instance, critical defects may require immediate intervention, such as halting the production line, whereas minor defects could be documented for further quality checks. Finally, the system generates actionable reports for operators, providing detailed insights into the type, location, and severity of each detected defect. These reports facilitate informed decision-making, streamline defect management processes, and ensure consistent product quality in real-time manufacturing environments [149].

## 4. Comparative Discussion on YOLO-Based Fabric Defect Detection

YOLO-based models have emerged as powerful real-time fabric defect detection tools due to their single-stage architecture, balance between speed and accuracy, and adaptability to various manufacturing scenarios. However, selecting the most appropriate YOLO variant depends on several factors, including precision requirements, computational constraints, and the nature of the fabric defects. Section 4 presents a focused comparative discussion on the performance and trade-offs of the YOLO model in fabric defect detection, drawing on Table 13 and Table 15 to highlight practical insights and challenges.

### 4.1. Performance Comparison of YOLO Variants in Fabric Defect Detection

Across various studies, YOLO-v5 and YOLO-v7 have demonstrated exceptional performance in fabric defect detection tasks, offering high detection accuracy with low latency. These models effectively handle diverse defect types, such as holes, stains, missing yarns, and misaligned weaves, due to their robust architectural features like Cross Stage Partial Networks (CSPNets) [150] and Path Aggregation Networks (PANets) [151]. YOLO-v5 is particularly well suited for industrial environments requiring lightweight deployment and fast inference on edge devices. Meanwhile, YOLO-v7 integrates E-ELAN for enhanced learning efficiency, making it particularly suitable for detecting complex and overlapping fabric defects.

Newer variants, from YOLO-v9 to YOLO-v11 [63,64,72], incorporate Transformer-based attention mechanisms, implement context-aware learning, and utilize hybrid loss functions such as CloU. These enhancements improve the detection of subtle and fine-grained defects in textured fabrics. However, such advancements come with higher computational costs, making them more suitable for GPU-based environments or post-inspection quality control rather than inline real-time detection systems. As reflected in Table 13, performance metrics, including mean Average Precision (mAP), detection accuracy, processing speed (FPS), and defect-specific detection rates, provide a comprehensive overview of how different YOLO variants adapt to various fabric defect detection tasks [152]. For instance, YOLO-based systems [113,115,117] have achieved accuracy levels of over 90% in detecting warp and weft defects, surpassing traditional methods such as Gabor filtering [153] and wavelet networks in terms of real-time capabilities [154] and defect localization precision [155].

### 4.2. Trade-Offs Between Speed and Accuracy

One of the most significant factors influencing model selection for fabric defect detection is the trade-off between speed and accuracy. Lightweight models such as YOLO-v5-n, YOLO-v6-n, and YOLO-v8-n [73,74,77] offer high inference speed with moderate accuracy, making them suitable for rapid screening or embedded systems with constrained resources. In contrast, larger models such as YOLO-v10-x and YOLO-v11-x [80,81] achieve higher accuracy, particularly in detecting complex or subtle fabric defects, but require high-performance GPUs and incur higher inference latency. As illustrated in Table 13, systems based on YOLO-v4 [113,117] and Mobile-YOLO [115] achieved high detection accuracies, often exceeding 90%. However, their suitability for real-time deployment varied depending on computational capacity and application constraints. Balancing detection performance with operational speed remains crucial in textile production lines, where bottlenecks caused by slow processing can disrupt manufacturing flow.

### 4.3. Role of Innovations in Enhancing Fabric Inspection

Several architectural innovations in recent YOLO models have significantly improved fabric defect detection performance and addressed practical challenges in real-world textile manufacturing. The implementation of advanced loss functions, such as Complete Intersection over Union (CIoU), has enhanced bounding box regression accuracy [156], especially for small or irregularly shaped defects commonly found in woven or knitted textiles [157,158]. Transformer-based attention mechanisms, particularly in YOLO-v9 to YOLO-v11, facilitate enhanced contextual feature extraction, enabling models to distinguish subtle defects from complex backgrounds. Innovations such as dynamic label assignment, adaptive scaling, and lightweight detection heads improve learning efficiency and deployment readiness in high-performance and edge-computing environments.

Despite these advancements and practical challenges highlighted in Table 15, key challenges in fabric defect detection include dataset imbalance, annotation costs, and scalability constraints, which remain obstacles to effective industrial implementation [14,16]. Scalability concerns also persist when integrating YOLO-based detection into high-speed production lines. To address these limitations, ongoing research is exploring the generation of synthetic data, semi-supervised learning, domain adaptation, and the deployment of edge AI. These techniques aim to maintain high accuracy and efficiency while reducing data dependency and adapting to diverse real-world fabric conditions.

Further advancements may focus on integrating YOLO models with edge computing platforms [159] and Internet of Things (IoT) frameworks [160]. Deploying lightweight YOLO variants on edge devices such as industrial cameras or embedded processors enables real-time defect detection directly at production lines, minimizing latency and reducing reliance on centralized computing systems [161]. IoT integration can facilitate seamless data collection and transmission, creating intelligent quality control ecosystems in textile manufacturing [162]. Moreover, the use of synthetic datasets generated by generative adversarial networks (GANs) [163] or simulation-based methods [164], combined with domain adaptation techniques [165], can enhance model generalization and reduce the burden of manual labeling. These strategies support robust, scalable, and adaptive fabric defect detection systems, which are better suited to the evolving demands of modern textile production environments.

## 5. Conclusions

This paper provides a comprehensive review of the YOLO object detection framework, tracing its evolution from YOLO-v1 to YOLO-v11 and assessing its application in fabric defect detection. This study highlights key advancements made across various YOLO versions, including the incorporation of Transformer-based attention mechanisms, multi-scale feature fusion, and adaptive learning techniques. These innovations have consistently enhanced the accuracy, speed, and generalization capabilities of YOLO models, making them increasingly suitable for real-world manufacturing settings. Earlier YOLO versions, YOLO-v1 to v3, focused primarily on real-time detection but encountered challenges in identifying minor and overlapping defects. Subsequent iterations, YOLO-v4 to v7, introduced improved feature extraction techniques, anchor-based optimizations, and advanced loss functions, resulting in notable enhancements in defect localization accuracy. More recent models, YOLO-v8 to v11, have embraced Transformer architectures, self-attention mechanisms, and adaptive resolution scaling to enhance defect detection performance, particularly in complex and cluttered fabric textures.

This review highlights the practical implications of YOLO models in industrial automation through comparative analysis. Implementing YOLO-based defect detection systems has demonstrated the potential to improve quality control, reduce production downtime, and lessen reliance on manual inspection. However, challenges remain, including limited datasets, difficulties with cross-domain generalization, and the significant computational demands associated with high-resolution fabric inspection. Tackling these challenges requires further research into synthetic dataset generation, federated learning for privacy-centric model training, and edge AI deployment for real-time defect detection in resource-constrained settings. Future research focuses on optimizing YOLO models for specific fabric defects by integrating domain-adaptive learning techniques and hybrid architectures that combine convolutional and Transformer-based networks. Developing standardized benchmark datasets tailored to the textile manufacturing sector will also be crucial for advancing this field. By linking advances in deep learning with industrial applicability, YOLO-based fabric defect detection systems can significantly transform next-generation quality assurance frameworks.

## Figures and Tables

**Table 1:** Summary of recent survey papers on fabric defect detection and their scope limitations.

Ref.	Year	Features	Drawbacks
Rasheed et al. [19]	2020	Review of traditional techniques: histogram-based, color-based, image segmentation, frequency domain, texture-based.	Limited coverage of recent deep learning methods. Sensitivity to variations in lighting and imaging conditions.
Czimmermann et al. [20]	2020	A detailed taxonomy of fabric defects, including both visible and palpable defects.	Limited discussion is provided on the application of deep learning models for specific types of defects.
Li et al. [21]	2021	Comparison of traditional and learning-based algorithms. Emphasis on practical implementation.	Limited coverage of the latest advancements in deep learning architectures, such as transformers and attention mechanisms.
Ahmad et al. [22]	2022	Focus on the advantages of deep learning for defect detection, such as feature learning.	High reliance on large, labeled datasets, which can be expensive and time-consuming to acquire.
Neethu et al. [23]	2023	Comparison of traditional and automated defect detection methods.	Limited discussion on the challenges and limitations of deep learning models in real-world industrial settings.
Jha et al. [24]	2023	Focus on deep CNN-based defect detection.	High reliance on large, labeled datasets and limited discussion on model interpretability and explainability.
Kulkarni et al. [25]	2024	Emphasis on proactive quality control and real-time defect detection.	Sensitivity to variations in image quality and lighting conditions.
Carrilho et al. [26]	2024	Comprehensive review of various defect detection methodologies, including traditional, spectral, model-based, and deep learning approaches.	Lack of standardized datasets for training and validation, as well as limited discussion on the challenges of applying deep learning models in the textile industry.
Hussain [27]	2024	Reviews all major YOLO versions (v1 to v8), providing a detailed analysis of their architectural evolution, training strategies, and performance metrics.	The application selection may not fully represent all possible uses of YOLO.
This study	2025	Comprehensive YOLO models (YOLO-v1 to YOLO-v11) for fabric defect detection. Focus on architectural advancements and real-world applications.	-

**Table 2:** Darknet-19 framework for YOLO-v2 [58].

Types	Filters	Size/Stride	Output
Convolutional	32	3 × 3	224 × 224
Maxpool	-	2 × 2/2	112 × 112
Convolutional	64	3 × 3	112 × 112
Maxpool	-	2 × 2/2	56 × 56
Convolutional	128	3 × 3	56 × 56
Convolutional	64	1 × 1	56 × 56
Convolutional	128	3 × 3	56 × 56
Maxpool		2 × 2/2	28 × 28
Convolutional	256	3 × 3	28 × 28
Convolutional	128	1 × 1	28 × 28
Convolutional	256	3 × 3	28 × 28
Maxpool	-	2 × 2/2	14 × 14
Convolutional	512	3 × 3	14 × 14
Convolutional	256	1 × 1	14 × 14
Convolutional	512	3 × 3	14 × 14
Convolutional	256	1 × 1	14 × 14
Convolutional	512	3 × 3	14 × 14
Maxpool	-	2 × 2/2	7 × 7
Convolutional	1024	3 × 3	7 × 7
Convolutional	512	3 × 3	7 × 7
Convolutional	1024	3 × 3	7 × 7
Convolutional	512	1 × 1	7 × 7
Convolutional	1024	3 × 3	7 × 7
Convolutional	1000	1 × 1	7 × 7
Avgpool		Global	1000
Softmax			

**Table 3:** YOLOv3 architecture [70].

Layer	Filters	Size	Repeats	Output Size
Image	-	-	-	416 × 416
Convolutional	32	3 × 3/1	1	416 × 416
Convolutional	64	3 × 3/2	1	208 × 208
Convolutional	32	1 × 1/1	Conv × 1	208 × 208
Convolutional	64	3 × 3/1	Conv × 1	208 × 208
Residual	-	-	Residual × 1	208 × 208
Convolutional	128	3 × 3/2	1	104 × 104
Convolutional	64	1 × 1/1	Conv × 2	104 × 104
Convolutional	128	3 × 3/1	Conv × 2	104 × 104
Residual	-	-	Residual × 2	104 × 104
Convolutional	256	3 × 3/2	1	52 × 52
Convolutional	128	1 × 1/1	Conv × 8	52 × 52
Convolutional	256	3 × 3/1	Conv × 8	52 × 52
Residual	-	-	Residual × 8	52 × 52
Convolutional	512	3 × 3/2	1	26 × 26
Convolutional	128	1 × 1/1	Conv × 8	26 × 26
Convolutional	512	3 × 3/1	Conv × 8	26 × 26
Residual	-	-	Residual × 8	26 × 26
Convolutional	1024	3 × 3/2	1	13 × 13
Convolutional	512	1 × 1/1	Conv × 4	13 × 13
Convolutional	1024	3 × 3/1	Conv × 4	13 × 13
Residual	-	-	Residual × 4	13 × 13

**Table 4:** YOLO-v4 architecture [71].

Layer	Filter	Size	Repeat	Output Size
Input Image	-	-	-	608 × 608
Conv	32	3 × 3/1	1	608 × 608
Conv	64	3 × 3/2	1	304 × 304
CSP Block	64	-	1	304 × 304
Conv	128	3 × 3/2	1	152 × 152
CSP Block	128	-	2	152 × 152
Conv	256	3 × 3/2	1	76 × 76
CSP Block	256	-	8	76 × 76
Conv	512	3 × 3/2	1	38 × 38
CSP Block	512	-	8	38 × 38
Conv	1024	3 × 3/2	1	19 × 19
SPP	-	-	1	19 × 19
CSP Block	512	-	4	19 × 19
YOLO Head	3 Scales	-	-	19 × 19

**Table 5:** Comparison of YOLO-v5 variants trained on the COCO dataset [73].

Models	Size (Pixels)	mAP50-95 (%)	Speed CPU ONNX (ms)	Speed A100 TensorRT (ms)	Params (M)	FLOPs (B)
YOLO-v5-n	640	34.3	73.6	1.06	2.6	7.7
YOLO-v5-s	640	43.0	120.7	1.27	9.1	24.0
YOLO-v5-m	640	49.0	233.9	1.86	25.1	64.2
YOLO-v5-l	640	52.2	408.4	2.50	53.2	135.0
YOLO-v5-x	640	53.2	763.3	3.81	97.2	246.4

**Table 6:** Comparison of YOLO-v6 variant training on the COCO dataset using an NVIDIA T4 [74].

Models	Size (Pixels)	mAP50-95 (%)	Speed A100 TensorRT (ms)	FPS	Params (M)	FLOPs (B)
YOLO-v6-n	640	37.5	1.17	1187	4.7	11.4
YOLO-v6-s	640	45.0	2.66	484	18.5	45.3
YOLO-v6-m	640	50.0	5.28	226	34.9	85.8
YOLO-v6-l	640	52.8	8.95	116	59.6	150.7

**Table 7:** Comparison of YOLO-v7 variant experiments on the COCO dataset [75].

Models	Size (Pixels)	FPS	mAP50-95 (%)	Params (M)	FLOPs (G)
YOLO-v7-Tiny	640	114	38.7	6.2	13.8
YOLO-v7	640	161	51.4	36.9	104.7
YOLO-v7-X	640	114	53.1	71.3	189.9
YOLO-v7-E6	1280	56	56.6	97.2	515.2
YOLO-v7-D6	1280	44	56.6	154.4	806.8

**Table 8:** Comparison of YOLO-v8 variant training on the COCO dataset [77].

Models	Size (Pixels)	mAP50-95 (%)	Speed CPU ONNX (ms)	Speed A100 TensorRT (ms)	Params (M)	FLOPs (B)
YOLO-v8-n	640	37.3	80.4	0.99	3.2	8.7
YOLO-v8-s	640	44.9	128.4	1.20	11.2	28.6
YOLO-v8-m	640	50.2	234.7	1.83	25.9	78.9
YOLO-v8-l	640	52.9	375.2	2.39	43.7	165.2
YOLO-v8-x	640	53.9	479.1	3.53	68.2	257.8

**Table 9:** The performance of YOLO-v9 on the COCO dataset [78].

Models	Size (Pixels)	mAP50-95 (%)	Speed CPU ONNX (ms)	Speed A100 TensorRT (ms)	Params (M)	FLOPs (B)
YOLO-v9-t	640	38.3	-	2.3	2.0	7.7
YOLO-v9-s	640	46.8	-	3.54	7.1	26.4
YOLO-v9-m	640	51.4	-	6.43	20.1	76.3
YOLO-v9-c	640	53.0	-	7.17	25.3	102.1
YOLO-v9-e	640	55.6	-	16.77	57.3	189.0

**Table 10:** Comparison of YOLO-v10 variant training on COCO dataset [80].

Models	Size (Pixels)	mAP50-95 (%)	Speed CPU ONNX (ms)	Speed A100 T4 TensorRT10 (ms)	Params (M)	FLOPs (B)
YOLO-v10-n	640	39.5	-	1.56	2.3	6.7
YOLO-v10-s	640	46.7	-	2.66	7.2	21.6
YOLO-v10-m	640	51.3	-	5.48	15.4	59.1
YOLO-v10-b	640	52.7	-	6.54	24.4	92.0
YOLO-v10-l	640	53.3	-	8.33	29.5	120.3
YOLO-v10-x	640	54.4	-	12.2	56.9	160.4

**Table 11:** Variant comparison of YOLO-v11 [81].

Models	Size (Pixels)	mAPVal50-95 (%)	Speed CPU ONNX (ms)	Speed T4 TensorRT10 (ms)	Params (M)	FLOPs (B)
YOLO-v11-n	640	39.5	56.1 ± 0.8	1.5 ± 0.0	2.6	6.5
YOLO-v11-s	640	47.0	90.0 ± 1.2	2.5 ± 0.0	9.4	21.5
YOLO-v11-m	640	51.5	183.2 ± 2.0	4.7 ± 0.1	20.1	68.0
YOLO-v11-l	640	53.4	238.6 ± 1.4	6.2 ± 0.1	25.3	86.9
YOLO-v11-x	640	54.7	462.8 ± 6.7	11.3 ± 0.2	56.9	194.9

**Table 12:** Strength and weakness comparison of YOLO variants.

Model	Strengths	Weakness
YOLO-v1	Fast performance, real-time detection	Lower accuracy compared to two-stage detectors
YOLO-v2	Better performance with anchor boxes	Still suffers from accuracy issues
YOLO-v3	Improved accuracy with Darknet-53 backbone	Increased complexity, slower than earlier versions
YOLO-v4	Adaptable with various heads and backbones	Less user-friendly
YOLO-v5	Faster training and more accuracy than YOLO-v3	Less adaptable than YOLO-v4
YOLO-v6	Faster computations and parameters	Less research and resource availability
YOLO-v7	Accuracy and speed improvements over YOLO-v6	Higher complexity, risk of overfitting
YOLO-v8	Lighter and faster compared to YOLO-v7	Case-specific optimizations
YOLO-v9	Reduced model size, ideal for real-time applications	Focuses too much on specific objects, ignoring the rest
YOLO-v10	Improvement in real-time detection and accuracy, building on YOLO-v9’s focus on reduced model size	Overfitting or performance drops when applied to highly diverse datasets
YOLO-v11	Expected to bring further advancements in accuracy and speed	Likely to have higher computational requirements.

**Table 13:** Summarizes the evolution of loss functions on improving fabric defect detection.

Model	Bounding Box Regression	Classification	Key Advantage
YOLO-v1	MSE	BCE	Simplistic and efficient for early-stage object detection, though less precise for bounding box localization.
YOLO-v2	Sum Squared Error	BCE	Introduced anchor boxes for better bounding box predictions, improving small object detection.
YOLO-v3	GIoU/DIoU	CE	Enhanced localization accuracy by considering the overlap and shape alignment of bounding boxes.
YOLO-v4	CIoU	BCE/Focal Loss	Improved localization precision with CIoU and reduced class imbalance using Focal Loss.
YOLO-v5	CIoU	Focal Loss	Balanced speed and accuracy with robust handling of overlapping objects and class imbalance.
YOLO-v6	CIoU/DFL	VariFocal Loss	Enhanced small object detection and class probability estimation with dynamic Focal Loss.
YOLO-v7	CIoU	BCE	Optimized for real-time performance with reduced computational overhead.
YOLO-v8	CIoU/DFL	CE	Improved computational efficiency and adaptability for varied image resolutions.
YOLO-v9	L1 Loss	BCE	Simplified loss function for faster convergence and efficient mobile deployment.
YOLO-v10	Coordinate loss/ confidence loss	CE	Enhanced accuracy for complex scenes with hybrid attention mechanisms.
YOLO-v11	IoU-based loss	BCE/Focal Loss + CloU	Achieved superior detection in dense environments by combining IoU-based precision with CloU’s adaptability.

**Table 14:** Summary of YOLO version improvements.

YOLO Variant	Tasks	Improvement	Results
YOLO-v1 [57]	OD, Basic Classification	Single-shot detection	Real-time speed, essential accuracy
YOLO-v2 [58]	OD, Improved Classification	Batch normalization, anchor boxes	Improved detection of small objects
YOLO-v3 [59]	OD, Multi-scale Detection	Multi-scale detection	Robust localization across scales
YOLO-v4 [60]	OD, Basic OD	SPP, mosaic augmentation	Enhanced generalization
YOLO-v5 [72]	OD, Basic Instance Segmentation (via custom modifications)	CSP network, auto-anchor learning	Optimized for real-time inspection
YOLO-v6 [61]	OD, Instance Segmentation	EfficientRep backbone	Improved detection of subtle defects
YOLO-v7 [62]	OD, Object Tracking, Instance Segmentation	Rep-PAN neck, anchor-free training	Effective for diverse fabrics
YOLO-v8 [76]	OD, Instance Segmentation, Classification, Oriented Detection, and Pose/Keypoint Detection	Anchor-free detection, modular design	High computational efficiency
YOLO-v9 [63]	OD, Instance Segmentation	Transformer-based attention mechanisms	Contextual understanding
YOLO-v10 [64]	OD	Hybrid attention mechanisms, adaptive scaling	Enhanced edge-device performance
YOLO-v11 [72]	OD, Instance Segmentation, Keypoint Estimation, Oriented Detection, Classification	Multi-scale attention, adaptive learning	Superior detection in varied settings

**Table 15:** Comparison of YOLO versions and its successors.

YOLO Version	Architecture (Backbone)	Neck	Anchor Boxes	Features	Framework
YOLO-v1	Darknet-24	-	Handpicked	1 × 1 convolutions, global average pooling, linear activation, and leaky ReLU.	Darknet
YOLO-v2	Darknet-19	-	Five anchor boxes using K-means	Batch normalization, high-resolution classifier, convolution with anchor boxes, dimension clusters, direct location prediction, fine-grained features, and multi-scale training.	Darknet
YOLO-v3	Darketnet-53	Feature Pyramid Networks	Night anchor boxes using clustering.	Independent Logistic classifiers, multi-scale training, and predictions.	Darknet
YOLO-v4	CSP-Darknet53	Path Aggregation Network	Night anchor boxes using clustering.	Spatial Pyramid Pooling (SPP), DropBlock regularization, Mish activation, ReLU6, class label smoothing, and cross mini-batch normalization.	Darknet
YOLO-v5	CSP-Darknet53	Spatial Pyramid Pooling—Fast (SPPF) and Cross Stage Partial (CSP) -PAN	Night anchor boxes using clustering.	Anchor-free split Ultralytics head: optimized accuracy–speed tradeoff and a variety of pre-trained models.	PyTorch
YOLO-v6	CSP-StackRep	Bi-directional Concatenation (Bic), Reparametrized (Rep-PAN)	Night anchor boxes using clustering.	Bi-directional concatenation (Bic) module, anchor-aided training (AAT) strategy, enhanced backbone and neck design, and self-distillation strategy.	PyTorch
YOLO-v7	Trainable bag-of-freebies	-	Night anchor boxes using clustering.	Model re-parameterization, dynamic label assignment, extended and compound scaling, and efficiency.	PyTorch
YOLO-v8	CSP-Darknet or customizable backbone	Path Aggregation Network (PANet)	Anchor-free with adaptive clustering	Improved computational efficiency, support for anchor-free detection, enhanced detection head, and compatibility with various tasks (e.g., segmentation).	PyTorch
YOLO-v9	EfficientNet-Lite	Bi-directional-FPN	Adaptive anchor boxes	Lightweight model design for mobile devices, knowledge distillation, post-processing optimization, and feature recalibration using the squeeze-and-excitation approach.	PyTorch
YOLO-v10	CSP-EfficientNet	Bi-directional Feature Pyramid (BiFPN)	Auto anchor learning	Hybrid attention mechanisms, Transformer-based detection refinement, global context embedding, and reduced latency for real-time applications.	PyTorch
YOLO-v11	Extended ResNet (E-ResNet)	Transformer-enhanced PANet	Dynamic anchor assignment	Integration of self-attention for long-range dependencies, depth-wise separable convolutions, feature fusion with Transformer blocks, and improved adaptability.	PyTorch

**Table 16:** Fabric defect detection systems and methods.

Paper	YOLO Version	Proposed Techniques	Accuracy
Guo et al. [87]	YOLO-v7	Deformable convolution, attention mechanisms, DCN-bottleneck module, MPCA module, K-means clustering for anchor boxes	mAP improved by 15.43% over the original YOLO-v7.
Yaohis et al. [111]	YOLO-v7-tiny	Partial convolution, dilated Spatial Pyramid Pooling fast cross-stage partial concat, attention mechanism, reduced parameters	9.55% mAP increase with 10.81% fewer parameters than YOLO-v7.
Yue et al. [112]	YOLO-v4	Data augmentation, anchor clustering, new prediction layers, attention mechanisms, optimized loss functions	12% improvement in tiny target AP and a 3% overall mAP increase.
Li et al. [113]	YOLO-v4	DBSCAN for anchor determination, enhanced feature extraction network, and fusion detection experiments	mAP of 98.97%, surpassing SSD (+7.67%), Faster R-CNN (+3.75%), and YOLO-v4-tiny (+10.82%).
Jing et al. [46]	YOLO-v3	Dimension clustering with K-means, integration of low- and high-level features, additional YOLO detection layer for multi-scale feature maps	Achieved 77.2% mAP.
Kawaguchi et al. [114]	YOLO	Real-time defect detection system for textile processing, streamlining detection and inspection for production workflows	Detects 1–2 mm defects with 60–70% accuracy.
Li et al. [115]	Mobile-YOLO	MobileNetV3 integration, cascaded network, attention mechanisms for improving detection of various defects	Achieved 99.4% accuracy, 16.8 million parameters, and 186 FPS.
Rasheed et al. [19]	-	Comprehensive review of automated fabric defect detection methods, comparison of manual vs. computerized methods, future research directions	-
Luo et al. [116]	YOLO-SCD	Lightweight detector, attention mechanisms, categorization of defect detection methods (structural, statistical, frequency domain, model-based analyses)	mAP improved by 8.49% to achieve 82.92%.
Zhang et al. [117]	YOLO-v4	Multi-channel fusion segmentation, impurity classification, and optimized YOLO-v4 for impurity detection in machine-picked seed cotton	The recognition rate is 94.1%.

**Table 17:** Datasets for fabric defect detection applications.

Datasets	# of Images	Resolutions	# of Classes	Year
Tianchi AI [121]	1000	513 × 513, 1536 × 1536	15	2018
TILDA [122]	896	416 × 416	4	2021
KTH-TIPS-I [123]	357	200 × 200	10	2004
KTH-TIPS-II [123]	481	200 × 200	11	2006
Brodatz Textures [124]	700	60 × 60, 512 × 512	9	-
AITEX Fabric [125]	245	4096 × 256	7	2023
TIDA 400 [126]	25,600	64 × 64	5	2024
HKU Fabric [127]	250	256 × 256	6	2013
Fabric Stain Dataset [128]	466	1920 × 1080, 1080 × 1920	5	-
Lusitano [129]	36,000	4096 × 1024	35	2024
ZJU-Leaper [130]	98,777	-	19	2020
YDFID-1 [131]	3830	512 × 512 × 3	19	2022

**Table 18:** Challenges in fabric detection.

YOLO Version	Key Features	Challenges in Fabric Detection	Improvements
YOLO-v1	Single-shot detection, coarse grid structure	Limited ability to detect small or densely packed defects.	Real-time speed but lower accuracy.
YOLO-v2	Anchor boxes, batch normalization	Improved detection of minor defects (e.g., small tears, stains) through higher-resolution imaging.	Enhanced accuracy and speed.
YOLO-v3	Multi-scale detection, FPN	Detect overlapping defects and complex textures by analyzing features at three granular levels.	20% improvement in mAP for small defects.
YOLO-v4	CSPDarknet, mosaic augmentation	Better generalization across varying lighting conditions and patterns.	Improved training efficiency and accuracy.
YOLO-v5	CSPNet, auto anchor learning	Optimized for real-time detection on high-speed production lines.	Reduced computational overhead.
YOLO-v6	EfficientRep backbone, RepPAN neck	Improved detection of subtle defects and enabled anchor-free training.	Enhanced speed and accuracy.
YOLO-v7	E-ELAN, dynamic label assignment	Better detection of diverse defects with improved scalability for high-speed environments.	Increased efficiency and accuracy.
YOLO-v8	Anchor-free detection, modular architecture	Enhanced adaptability and computational efficiency for detecting complex fabric defects, such as irregular textures.	High efficiency and flexibility.
YOLO-v9	Transformer-based attention mechanisms	Better contextual understanding for detecting defects in diverse textures and lighting conditions.	Improved accuracy in challenging environments.
YOLO-v10	Hybrid attention mechanisms, adaptive scaling	Improved precision for detecting subtle and overlapping defects in real-time scenarios.	Enhanced edge-device performance.
YOLO-v11	Multi-scale attention, adaptive learning	Superior detection of defects across varied lighting and fabric types.	Advanced real-time performance.

## Data Availability

Data are not available on request due to restrictions, e.g., privacy or ethical.

## Abbreviations

The following abbreviations are used in this manuscript:

**Abbreviations**	**Description**
AAT	Anchor-Aided Training
AI	Artificial intelligence
BiCSP	Bi-directional Cross-Stage Partial
BiFPN	Bi-directional Feature Pyramid
BCE	Binary Cross Entropy
CPU	Central Processing Unit
CIoU	Complete IoU
CNNs	Convolutional neural networks
CE	Cross Entropy
CSP	Cross-Stage Partial
CSPNet	Cross-Stage Partial Network
DBSCAN	Density-Based Spatial Clustering of Applications with Noise
DFL	Distribution Focal Loss
FPN	Feature Pyramid Network
FLOPs	Floating-Point Operations Per Second
GANs	Generative Adversarial Networks
GPUs	Graphics processing unit
IoT	Internet of Things
mAP	mean Average Precision
MSE	Mean Squared Error
NMS	non-maximum suppression
OD	Object detection
PANets	Path Aggregation Networks
Rep-PAN	Reparametrized
SIoU	Skew Intersection over Union
SAM	Spatial Attention Module
SPP	Spatial Pyramid Pooling
SPPF	Spatial Pyramid Pooling—Fast
SVMs	support vector machines
YOLO	You Only Look Once

## References

[1] Hassan S.A., Beliatis M.J., Radziwon A., Menciassi A., Oddo C.M. (2024). Textile Fabric Defect Detection Using Enhanced Deep Convolutional Neural Network with Safe Human–Robot Collaborative Interaction. Electronics.

[2] Mahmud T., Sikder J., Chakma R.J., Fardoush J. Fabric defect detection system. Proceedings of the 3rd International Conference on Intelligent Computing and Optimization (ICO).

[3] Ngan H.Y., Pang G.K., Yung N.H. (2011). Automated fabric defect detection—A review. Image Vis. Comput..

[4] Diwan T., Anirudh G., Tembhurne J.V. (2023). Object detection using YOLO: Challenges, architectural successors, datasets and applications. Multimed. Tools Appl..

[5] Liu Q., Wang C., Li Y., Gao M., Li J. (2022). A fabric defect detection method based on deep learning. IEEE Access..

[6] Kaur R., Singh S. (2023). A comprehensive review of object detection with deep learning. Digit. Signal Process..

[7] Liu C., Tao Y., Liang J., Li K., Chen Y. Object detection based on YOLO network. Proceedings of the IEEE 4th Information Technology and Mechatronics Engineering Conference (ITOEC).

[8] Mahajan P.M., Kolhe S.R., Patil P.M. (2009). A review of automatic fabric defect detection techniques. Adv. Comput. Res..

[9] Zhao Z.Q., Zheng P., Xu S.T., Wu X. (2019). Object detection with deep learning: A review. IEEE Trans. Neural Netw. Learn. Syst..

[10] Ali M.L., Zhang Z. (2024). The YOLO Framework: A Comprehensive Review of Evolution, Applications, and Benchmarks in Object Detection. Computers.

[11] Wang X., Georganas N.D., Petriu E.M. (2010). Fabric texture analysis using computer vision techniques. EEE Trans. Instrum. Meas..

[12] Nasim M., Mumtaz R., Ahmad M., Ali A. (2024). Fabric Defect Detection in Real World Manufacturing Using Deep Learning. Information.

[13] Ahmad T., Ma Y., Yahya M., Ahmad B., Nazir S., Haq A.U. (2020). Object detection through modified YOLO neural network. Sci. Program..

[14] Rane N. (2023). YOLO and Faster R-CNN Object Detection for Smart Industry 4.0 and Industry 5.0: Applications, Challenges, and Opportunities. https://papers.ssrn.com/sol3/papers.cfm?abstract_id=4624206.

[15] Divyadevi R., Kumar B.V. Survey of automated fabric inspection in textile industries. Proceedings of the International Conference on Computer Communication and Informatics (ICCCI).

[16] Hussain M. (2023). YOLO-v1 to YOLO-v8, the Rise of YOLO and Its Complementary Nature toward Digital Manufacturing and Industrial Defect Detection. Machines.

[17] Sujee R., Shanthosh D., Sudharsun L. Fabric defect detection using YOLOv2 and YOLO v3 tiny. Proceedings of the Computational Intelligence in Data Science: Third IFIP TC 12 International Conference (ICCIDS).

[18] Malaca P., Rocha L.F., Gomes D., Silva J., Veiga G. (2019). Online inspection system based on machine learning techniques: Real case study of fabric textures classification for the automotive industry. J. Intell. Manuf..

[19] Rasheed A., Zafar B., Rasheed A., Ali N., Sajid M., Dar S.H., Habib U., Shehryar T., Mahmood M.T. (2020). Fabric defect detection using computer vision techniques: A comprehensive review. Math. Probl. Eng..

[20] Czimmermann T., Ciuti G., Milazzo M., Chiurazzi M., Roccella S., Oddo C.M., Dario P. (2020). Visual-Based Defect Detection and Classification Approaches for Industrial Applications—A Survey. Sensors.

[21] Li C., Li J., Li Y., He L., Fu X., Chen J. (2021). Fabric defect detection in textile manufacturing: A survey of the state of the art. Secur. Commun. Netw..

[22] Ahmad H.M., Rahimi A. (2022). Deep learning methods for object detection in smart manufacturing: A survey. J. Manuf. Syst..

[23] Neethu Pavithran C., Binoy K.P. Fabric Defect Detection: A Review. Proceedings of the International Conference Emerging Trends in Engineering (YUKTHI 2023).

[24] Jha S.B., Babiceanu R.F. (2023). Deep CNN-based visual defect detection: Survey of current literature. Comput. Ind..

[25] Kulkarni M., Cholke P.C., Vasmatkar P., Tambe S., Anuse S., Telgote S. Fabric Defect Detection System Using Image Processing. Proceedings of the 1st International Conference on Cognitive, Green and Ubiquitous Computing (IC-CGU).

[26] Carrilho R., Yaghoubi E., Lindo J., Hambarde K., Proença H. (2024). Toward Automated Fabric Defect Detection: A Survey of Recent Computer Vision Approaches. Electronics.

[27] Hussain M. (2024). Yolov1 to v8: Unveiling each variant—A comprehensive review of yolo. IEEE Access.

[28] Gupta A.K., Seal A., Prasad M., Khanna P. (2020). Salient Object Detection Techniques in Computer Vision—A Survey. Entropy.

[29] Pathak A.R., Pandey M., Rautaray S. (2018). Application of deep learning for object detection. Procedia Comput. Sci..

[30] Yao R., Lin G., Xia S., Zhao J., Zhou Y. (2020). Video object segmentation and tracking: A survey. ACM Trans. Inte. Syst. Tech. TIST.

[31] Wang C., Yang H., Bartz C., Meinel C. Image captioning with deep bidirectional LSTMs. Proceedings of the 24th ACM International Conference on Multimedia.

[32] Meimetis D., Daramouskas I., Perikos I., Hatzilygeroudis I. (2023). Real-time multiple object tracking using deep learning methods. Neural Comput. Appl..

[33] Bachute M.R., Subhedar J.M. (2021). Autonomous driving architectures: Insights of machine learning and deep learning algorithms. Mach. Learn. Appl..

[34] She Q., Feng F., Hao X., Yang Q., Lan C., Lomonaco V., Shi X., Wang Z., Guo Y., Zhang Y. Openloris-object: A robotic vision dataset and benchmark for lifelong deep learning. Proceedings of the International Conference on Robotics and Automation (ICRA).

[35] Zahrawi M., Shaalan K. (2023). Improving video surveillance systems in banks using deep learning techniques. Sci. Rep..

[36] Fukushima K. (1980). Neocognitron: A self-organizing neural network model for a mechanism of pattern recognition unaffected by shift in position. Biol. Cybern..

[37] Lee S.M., Yoon S.M., Cho H. Human activity recognition from accelerometer data using Convolutional Neural Network. Proceedings of the International Conference on Big Data and Smart Computing (BIGCOMP).

[38] Jmour N., Zayen S., Abdelkrim A. Convolutional neural networks for image classification. Proceedings of the International Conference on Advanced Systems and Electric Technologies (ICASET).

[39] Wu Y.C., Yin F., Liu C.L. (2017). Improving handwritten Chinese text recognition using neural network language models and convolutional neural network shape models. Pattern Recognit..

[40] Coşkun M., Uçar A., Yildirim Ö., Demir Y. Face recognition based on convolutional neural network. Proceedings of the International Conference on Modern Electrical and Energy Systems (MEES).

[41] Teng S., Kim J.-Y., Jeon S., Gil H.-W., Lyu J., Chung E.H., Kim K.S., Nam Y. (2024). Analyzing Optimal Wearable Motion Sensor Placement for Accurate Classification of Fall Directions. Sensors.

[42] Park B., Lu R. (2015). Hyperspectral Imaging Technology in Food and Agriculture.

[43] Long Y., Gong Y., Xiao Z., Liu Q. (2017). Accurate object localization in remote sensing images based on convolutional neural networks. IEEE Trans. Geos. Remo. Sens..

[44] Mak K.L., Peng P., Yiu K.F.C. (2009). Fabric defect detection using morphological filters. Image Vis. Comput..

[45] Liu B., Wang H., Cao Z., Wang Y., Tao L., Yang J., Zhang K. (2024). PRC-Light YOLO: An Efficient Lightweight Model for Fabric Defect Detection. Appl. Sci..

[46] Jing J., Zhuo D., Zhang H., Liang Y., Zheng M. (2020). Fabric defect detection using the improved YOLOv3 model. J. Eng. Fibers Fabr..

[47] Liu B., Zhao W., Sun Q. Study of object detection based on Faster R-CNN. Proceedings of the Chinese Automation Congress (CAC).

[48] Carranza-García M., Torres-Mateo J., Lara-Benítez P., García-Gutiérrez J. (2021). On the Performance of One-Stage and Two-Stage Object Detectors in Autonomous Vehicles Using Camera Data. Remote Sens..

[49] Ren S., He K., Girshick R., Sun J. (2016). Faster R-CNN: Towards real-time object detection with region proposal networks. IEEE Trans. Patte. Anal. Mach. Intel..

[50] Soviany P., Ionescu R.T. Optimizing the trade-off between single-stage and two-stage deep object detectors using image difficulty prediction. Proceedings of the 20th International Symposium on Symbolic and Numeric Algorithms for Scientific Computing (SYNASC).

[51] Zhang J., Liu M., Shen D. (2017). Detecting anatomical landmarks from limited medical imaging data using two-stage task-oriented deep neural networks. IEEE Trans. Image Process..

[52] Sapkota R., Qureshi R., Flores-Calero M., Badgujar C., Nepal U., Poulose A., Zeno P., Bhanu Prakash Vaddevolu U., Yan P., Karkee M. (2024). Yolov10 to Its Genesis: A Decadal and Comprehensive Review of the You Only Look Once Series. https://papers.ssrn.com/sol3/papers.cfm?abstract_id=4874098.

[53] Gündüz M.Ş., Işık G. (2023). A new YOLO-based method for real-time crowd detection from video and performance analysis of YOLO models. J. Real-Time Image Process..

[54] Zheng C. (2023). Stack-YOLO: A friendly-hardware real-time object detection algorithm. IEEE Access.

[55] Zhang Z. (2023). Drone-YOLO: An Efficient Neural Network Method for Target Detection in Drone Images. Drones.

[56] Ma C., Fu Y., Wang D., Guo R., Zhao X., Fang J. (2023). YOLO-UAV: Object detection method of unmanned aerial vehicle imagery based on efficient multi-scale feature fusion. IEEE Access.

[57] Redmon J. You only look once: Unified, real-time object detection. Proceedings of the IEEE conference on computer vision and pattern recognition.

[58] Redmon J., Farhadi A. YOLO9000: Better, faster, stronger. Proceedings of the IEEE Conference on Computer Vision and Pattern Recognition.

[59] Farhadi A., Redmon J. (2018). Yolov3: An incremental improvement. Comput. Vis. Pattern Recognit..

[60] Bochkovskiy A., Wang C.Y., Liao H.Y.M. (2020). Yolov4: Optimal speed and accuracy of object detection. arXiv.

[61] Li C., Li L., Jiang H., Weng K., Geng Y., Li L., Ke Z., Li Q., Cheng M., Nie W. (2022). YOLOv6: A single-stage object detection framework for industrial applications. arXiv.

[62] Wang C.Y., Bochkovskiy A., Liao H.Y.M. YOLOv7: Trainable bag-of-freebies sets new state-of-the-art for real-time object detectors. Proceedings of the IEEE/CVF Conference on Computer Vision and Pattern Recognition, Vancouver Convention Center.

[63] Wang C.Y., Yeh I.H., Mark Liao H.Y. Yolov9: Learning what you want to learn using programmable gradient information. Proceedings of the European Conference on Computer Vision.

[64] Wang A., Chen H., Liu L., Chen K., Lin Z., Han J., Ding G. (2024). Yolov10: Real-time end-to-end object detection. arXiv.

[65] Adarsh P., Rathi P., Kumar M. YOLO v3-Tiny: Object Detection and Recognition using one stage improved model. Proceedings of the 6th International Conference on Advanced Computing and Communication Systems (ICACCS).

[66] Kang C.H., Kim S.Y. (2023). Real-time object detection and segmentation technology: An analysis of the YOLO algorithm. JMST Adv..

[67] Everingham M., Van Gool L., Williams C.K., Winn J., Zisserman A. (2010). The pascal visual object classes (voc) challenge. Proc. Int. J. Comput. Vis..

[68] Lin T.Y., Maire M., Belongie S., Hays J., Perona P., Ramanan D., Dollár P., Zitnick C.L. Microsoft coco: Common objects in context. Proceedings of the Computer Vision–ECCV 2014: 13th European Conference.

[69] Sinaga K.P., Yang M.S. (2020). Unsupervised K-means clustering algorithm. IEEE Access.

[70] DEV Community Is a Community of 2,921,747 Amazing Developers. https://dev.to/afrozchakure/all-you-need-to-know-about-yolo-v3-you-only-look-once-e4m.

[71] Geetha A.S. (2025). YOLOv4: A Breakthrough in Real-Time Object Detection. arXiv.

[72] Ultralytics Home Page. https://www.ultralytics.com/.

[73] Ultralytics Home Page. https://docs.ultralytics.com/models/yolov5/#performance-metrics.

[74] Ultralytics Home Page. https://docs.ultralytics.com/compare/yolov10-vs-yolov6/#model-comparison-table.

[75] Ultralytics Home Page. https://docs.ultralytics.com/models/yolov7/.

[76] Reis D., Kupec J., Hong J., Daoudi A. (2023). Real-time flying object detection with YOLOv8. arXiv.

[77] Ultralytics Home Page. https://docs.ultralytics.com/models/yolov8/#performance-metrics.

[78] Ultralytics Home Page. https://docs.ultralytics.com/compare/yolo11-vs-yolov9/#yolov9-efficiency-and-accuracy-innovations.

[79] COCO Dataset. https://github.com/ultralytics/ultralytics/blob/main/ultralytics/cfg/datasets/coco.yaml.

[80] Ultralytics Home Page. https://docs.ultralytics.com/compare/yolov10-vs-yolov6/#ideal-use-cases-for-yolov6-30.

[81] Ultralytics Home Page. https://docs.ultralytics.com/models/yolo11/#performance-metrics.

[82] Ye K., Gao Y., Ye X., Ye A. DC-YOLO: Improved YOLOv7 Based on Deformable Convolution and Attention Mechanism for Fabric Defect Detection. Proceedings of the Eleventh International Conference on Advanced Cloud and Big Data (CBD).

[83] Sekharamantry P.K., Melgani F., Malacarne J. (2023). Deep Learning-Based Apple Detection with Attention Module and Improved Loss Function in YOLO. Remote Sens..

[84] Du J. Understanding of object detection based on CNN family and YOLO. Proceedings of the International Conference on Machine Vision and Information Technology (CMVIT).

[85] Huang S., He Y., Chen X.A. M-YOLO: A Nighttime Vehicle Detection Method Combining Mobilenet v2 and YOLO v3. Proceedings of the 2nd International Conference on Computer Information and Big Data Applications.

[86] Zheng Z., Wang P., Liu W., Li J., Ye R., Ren D. Distance-IoU loss: Faster and better learning for bounding box regression. Proceedings of the AAAI Conference on Artificial Intelligence.

[87] Guo C., Zheng S., Cheng G., Zhang Y., Ding J. (2023). An improved YOLO v4 used for grape detection in unstructured environment. Front. Plant Sci..

[88] Liu Z., Wang S. (2019). Broken corn detection based on an adjusted YOLO with focal loss. IEEE Access.

[89] Maji D., Nagori S., Mathew M., Poddar D. Yolo-pose: Enhancing yolo for multi-person pose estimation using object keypoint similarity loss. Proceedings of the IEEE/CVF Conference on Computer Vision and Pattern Recognition.

[90] Saenprasert W., Tun E.E., Hajian A., Ruangsang W., Aramvith S. Yolo for small objects in aerial imagery: A performance evaluation. Proceedings of the 21st International Joint Conference on Computer Science and Software Engineering (JCSSE).

[91] Zou Z., Chen K., Shi Z., Guo Y., Ye J. (2023). Object detection in 20 years: A survey. Proc. IEEE.

[92] Zhang Y., Li L., Chun C., Wen Y., Xu G. (2024). Multi-scale feature adaptive fusion model for real-time detection in complex citrus orchard environments. Comput. Electron. Agric..

[93] Ribés A., Benchekroun N., Delagnes T. (2024). A Fast Learning-Based Surrogate of Electrical Machines using a Reduced Basis. arXiv.

[94] Cheng L. (2024). A Highly robust helmet detection algorithm based on YOLO V8 and Transformer. IEEE Access.

[95] Onthoni A.I., Sahoo P.K. Instance Segmentation based Object Detection with Enhanced Path Aggregation Network. Proceedings of the IEEE Latin American Conference on Computational Intelligence (LA-CCI).

[96] Chen J., Liu H., Zhang Y., Zhang D., Ouyang H., Chen X. (2022). A Multiscale Lightweight and Efficient Model Based on YOLOv7: Applied to Citrus Orchard. Plants.

[97] Nguyen D.T., Nguyen T.N., Kim H., Lee H.J. (2019). A high-throughput and power-efficient FPGA implementation of YOLO CNN for object detection. IEEE Trans. Very Large Scale Integr. VLSI Syst..

[98] Luo G., Zhou Y., Jin L., Sun X., Ji R. (2023). Towards end-to-end semi-supervised learning for one-stage object detection. arXiv.

[99] Yuan D., Geng G., Shu X., Liu Q., Chang X., He Z., Shi G. (2024). Self-supervised discriminative model prediction for visual tracking. Neural Comput. Appl..

[100] Liang S., Wu H., Zhen L., Hua Q., Garg S., Kaddoum G., Hassan M.M., Yu K. (2022). Edge YOLO: Real-time intelligent object detection system based on edge-cloud cooperation in autonomous vehicles. IEEE Trans. Intell. Transp. Syst..

[101] Van Engelen J.E., Hoos H.H. (2020). A survey on semi-supervised learning. Mach. Learn..

[102] Tong L., Wong W.K., Kwong C.K. (2017). Fabric defect detection for apparel industry: A nonlocal sparse representation approach. IEEE Access.

[103] Sobirov M., Sharibaev N., Kayumov A., Musayev K. Method of assessment of structural properties of knitted fabrics based on image analysis. Proceedings of the International Scientific Conference on Green Energy.

[104] Carrilho R., Hambarde K.A., Proença H. (2024). A Novel Dataset for Fabric Defect Detection: Bridging Gaps in Anomaly Detection. Appl. Sci..

[105] Xu C., Li W., Cui X., Wang Z., Zheng F., Zhang X., Chen B. (2024). Scarcity-GAN: Scarce data augmentation for defect detection via generative adversarial nets. Neurocomputing.

[106] Zhao Z., Ma X., Yang X., Wang F. Research on Fabric Defect Detection Algorithm Based on Improved YOLOv5. Proceedings of the IEEE 7th Advanced Information Technology, Electronic and Automation Control Conference (IAEAC).

[107] Liu J., Long X., Jiang C., Liao W. (2024). Multi-feature vision transformer for automatic defect detection and quantification in composites using thermography. NDT E Int..

[108] Zhao Y., Liu Q., Su H., Zhang J., Ma H., Zou W., Liu S. (2024). Attention-based Multi-scale Feature Fusion for Efficient Surface Defect Detection. IEEE Trans. Instr. Meas..

[109] Bai D., Li G., Jiang D., Yun J., Tao B., Jiang G., Sun Y., Ju Z. (2024). Surface defect detection methods for industrial products with imbalanced samples: A review of progress in the 2020s. Eng. Appl. Artif. Intell..

[110] Rodriguez-Vazquez J., Prieto-Centeno I., Fernandez-Cortizas M., Perez-Saura D., Molina M., Campoy P. (2024). Real-Time Object Detection for Autonomous Solar Farm Inspection via UAVs. Sensors.

[111] Yaohui Z., Jia R., Yu L. (2024). Yolov7-Tinier: Towards High-Precision and Lightweight Detection of Fabric Defects in Textile Plant. Fibers Polym..

[112] Yue X., Wang Q., He L., Li Y., Tang D. (2022). Research on Tiny Target Detection Technology of Fabric Defects Based on Improved YOLO. Appl. Sci..

[113] Li Y., Song L., Cai Y., Fang Z., Tang M. (2024). Research on fabric surface defect detection algorithm based on improved Yolo_v4. Sci. Rep..

[114] Kawaguchi M., Morimitsu Y., Watanabe R., Ikuta C., Ishii N., Umeno M. Fabric Defect Detection System using YOLO. Proceedings of the 14th IIAI International Congress on Advanced Applied Informatics (IIAI-AAI).

[115] Li J., Kang X. (2024). Mobile-YOLO: An accurate and efficient three-stage cascaded network for online fiberglass fabric defect detection. Eng. Appl. Artif. Intell..

[116] Luo X., Ni Q., Tao R., Shi Y. (2023). A lightweight detector based on attention mechanism for fabric defect detection. IEEE Access.

[117] Zhang C., Li T., Zhang W. (2022). The Detection of Impurity Content in Machine-Picked Seed Cotton Based on Image Processing and Improved YOLO V4. Agronomy.

[118] Wan Y., Wang H., Lu L., Lan X., Xu F., Li S. (2024). An Improved Real-Time Detection Transformer Model for the Intelligent Survey of Traffic Safety Facilities. Sustainability.

[119] Vaidwan H., Seth N., Parihar A.S., Singh K. A study on transformer-based object detection. Proceedings of the International Conference on Intelligent Technologies (CONIT).

[120] Usamentiaga R., Lema D.G., Pedrayes O.D., Garcia D.F. (2022). Automated surface defect detection in metals: A comparative review of object detection and semantic segmentation using deep learning. IEEE Trans. Ind. Appl..

[121] Tianchi AI Dataset. https://tianchi.aliyun.com/dataset/79336.

[122] TILDA Dataset. https://universe.roboflow.com/irvin-andersen/tilda-fabric/dataset/2.

[123] KTH-TIPS-I Dataset. https://www.csc.kth.se/cvap/databases/kth-tips/index.html.

[124] Brodatz Textures Dataset. https://www.ux.uis.no/~tranden/brodatz.html.

[125] AITEX Fabric Dataset. https://www.kaggle.com/datasets/nexuswho/aitex-fabric-image-database.

[126] TIDA 400 Dataset. https://www.kaggle.com/datasets/angelolmg/tilda-400-64x64-patches.

[127] HKU Fabric Dataset. https://ytngan.wordpress.com/codes/.

[128] Fabric Stain Dataset. https://www.kaggle.com/datasets/priemshpathirana/fabric-stain-dataset.

[129] Lusitano Dataset. https://kailashhambarde.github.io/Lusitano/#dataset.

[130] ZJU-Leaper Dataset. http://www.qaas.zju.edu.cn/zju-leaper/.

[131] YDFID-1 Dataset. https://github.com/ZHW-AI/YDFID-1/blob/main/README_ENG.md.

[132] Mak K.L., Peng P. (2008). An automated inspection system for textile fabrics based on Gabor filters. Robot. Comput.-Integr. Manuf..

[133] Jiang J., Jin Z., Wang B., Ma L., Cui Y. (2020). A Sobel operator combined with patch statistics algorithm for fabric defect detection. KSII Trans. Internet Inf. Syst..

[134] Zhu L., Spachos P. (2021). Support vector machine and YOLO for a mobile food grading system. Internet Things.

[135] Bhavatarini N., Akash B.N., Avinash A.R., Akshay H.M. Object detection and classification of hyperspectral images using K-NN. Proceedings of the Second International Conference on Electrical, Electronics, Information and Communication Technologies (ICEEICT).

[136] Jun X., Wang J., Zhou J., Meng S., Pan R., Gao W. (2021). Fabric defect detection based on a deep convolutional neural network using a two-stage strategy. Text. Res. J..

[137] Rani S., Ghai D., Kumar S. (2022). Object detection and recognition using contour based edge detection and fast R-CNN. Multimed. Tools Appl..

[138] Avudaiamal R., Subramaniakuppusamy K., Christopher N.S. YOLO: Roof material detection using aerial imagery. Proceedings of the International Conference on Computing and Data Science (ICCDS).

[139] Kailasam K., Singh J., Gopika G.S., Sreekrishna M., Supriya S. (2024). Supriya, Fabric Defect Detection using Deep Learning. Proceedings of the 3rd International Conference on Sentiment Analysis and Deep Learning (ICSADL).

[140] Saberironaghi A., Ren J., El-Gindy M. (2023). Defect Detection Methods for Industrial Products Using Deep Learning Techniques: A Review. Algorithms.

[141] Wang C., Zhou Z., Chen Z. (2022). An enhanced YOLOv4 model with self-dependent attentive fusion and component randomized mosaic augmentation for metal surface defect detection. IEEE Access.

[142] Han J., Zhang Z., Gao X., Li K., Kang X. Research on negative obstacle detection method based on image enhancement and improved anchor box YOLO. Proceedings of the IEEE International Conference on Mechatronics and Automation (ICMA).

[143] Sohaib M., Arif M., Kim J.-M. (2024). Evaluating YOLO Models for Efficient Crack Detection in Concrete Structures Using Transfer Learning. Buildings.

[144] Dai Y., Liu W., Wang H., Xie W., Long K. (2022). Yolo-former: Marrying yolo and transformer for foreign object detection. IEEE Trans. Instrum. Meas..

[145] Misra D., Nalamada T., Arasanipalai A.U., Hou Q. Rotate to attend: Convolutional triplet attention module. Proceedings of the IEEE/CVF Winter Conference on Applications of Computer Vision.

[146] Yang L., Zheng Z., Wang J., Song S., Huang G., Li F. (2023). Adadet: An adaptive object detection system based on early-exit neural networks. IEEE Trans. Cogn. Dev. Syst..

[147] Lee J., Hwang K.I. (2022). YOLO with adaptive frame control for real-time object detection applications. Multimed. Tools Appl..

[148] Guo Q., Wang C., Xiao D., Huang Q. (2021). An Enhanced Insect Pest Counter Based on Saliency Map and Improved Non-Maximum Suppression. Insects.

[149] Jain S., Seth G., Paruthi A., Soni U., Kumar G. (2022). Synthetic data augmentation for surface defect detection and classification using deep learning. J. Intell. Manuf..

[150] Islam M.R., Zamil M.Z.H., Rayed M.E., Kabir M.M., Mridha M.F., Nishimura S., Shin J. (2024). Deep Learning and Computer Vision Techniques for Enhanced Quality Control in Manufacturing Processes. IEEE Access.

[151] Wang C.Y., Bochkovskiy A., Liao H.Y.M. Scaled-yolov4: Scaling cross stage partial network. Proceedings of the IEEE/cvf Conference on Computer Vision and Pattern Recognition.

[152] Zhao W., Syafrudin M., Fitriyani N.L. (2023). CRAS-YOLO: A novel multi-category vessel detection and classification model based on YOLOv5s algorithm. IEEE Access.

[153] Xu H., Liu C., Duan S., Ren L., Cheng G., Hao B. (2023). A Fabric Defect Segmentation Model Based on Improved Swin-Unet with Gabor Filter. Appl. Sci..

[154] Wu J., Xu X., Liu C., Deng C., Shao X. (2021). Lamb wave-based damage detection of composite structures using deep convolutional neural network and continuous wavelet transform. Compos. Struct..

[155] Huang Y., Jing J., Wang Z. (2021). Fabric defect segmentation method based on deep learning. IEEE Trans. Instrum. Meas..

[156] Cheng L., Yi J., Chen A., Zhang Y. (2023). Fabric defect detection based on separate convolutional UNet. Multimed. Tools Appl..

[157] Meng S., Pan R., Gao W., Yan B., Peng Y. (2022). Automatic recognition of woven fabric structural parameters: A review. Artif. Intell. Rev..

[158] Zhao B., Dong Z., Cong H. (2022). A wearable and fully-textile capacitive sensor based on flat-knitted spacing fabric for human motions detection. Sens. Actuators A Phys..

[159] Ros S., Tam P., Song I., Kang S., Kim S. (2024). A survey on state-of-the-art experimental simulations for privacy-preserving federated learning in intelligent networking. Electron. Res. Arch..

[160] Tam P., Kim S. (2024). Graph-Based Deep Reinforcement Learning in Edge Cloud Virtualized O-RAN for Sharing Collaborative Learning Workloads. IEEE Trans. Netw. Sci. Eng..

[161] Nguyen T., Nguyen H., Gia T.N. (2024). Exploring the integration of edge computing and blockchain IoT: Principles, architectures, security, and applications. J. Netw. Comput. Appl..

[162] Li J., Wu R., Zhang S., Chen Y., Dong Z. (2023). FASCNet: An Edge-computational Defect Detection Model for Industrial Parts. IEEE Internet Things J..

[163] Espinàs Salla M.D.M. (2021). Data Analysis and GAN-Based Anonymization of a Database of Lung Cancer Patients. Bachelor’s Thesis.

[164] Peres R.S., Guedes M., Miranda F., Barata J. (2021). Simulation-based data augmentation for the quality inspection of structural adhesive with deep learning. IEEE Access.

[165] Frazier R., Zambrano F., Pawlak J.J., Gonzalez R. (2022). Methods to assess and control dusting and linting in the paper industry: A review. Int. J. Adv. Manuf. Technol..

